# Analysis of global, regional, and national burdens of neonatal encephalopathy from 1990 to 2021: insights from the Global Burden of Disease Study 2021

**DOI:** 10.3389/fpubh.2025.1627448

**Published:** 2025-10-08

**Authors:** Shujun Tan, Shala Mageziyi, Li Long, Naiyiman Dilimulati, Zhang Hui, Nuerya Rejiafu

**Affiliations:** Neonatal Center, Xinjiang Hospital of Beijing Children’s Hospital, Children’s Hospital of Xinjiang Uygur Autonomous Region, Urumqi, China

**Keywords:** neonatal encephalopathy, disease burden, sociodemographic index, prediction, global burden of disease

## Abstract

**Background:**

The Sustainable Development Goal 3.2 urges nations to reduce neonatal mortality rates to no more than 12 deaths per 1,000 live births by 2030. Neonatal encephalopathy (NE), the third leading cause of under-five mortality, significantly impacts global neonatal death rates and long-term health. A comprehensive assessment is essential to inform public health strategies. This study aims to analyze the burden of Neonatal Encephalopathy on global, regional, and national levels.

**Methods:**

Data on the age-standardized mortality rate (ASMR) and age-standardized incidence rates (ASIR) for NE from 1990 to 2021 were obtained from the Global Burden of Disease Study (GBD) 2021. To evaluate the changes in ASIR and ASMR associated with the burden of NE, estimated annual percentage changes (EAPC) and overall percentage changes were calculated. Furthermore, the relationship between disease burden and the Sociodemographic Index (SDI) was analyzed, along with predictions generated using autoregressive integrated moving average (ARIMA) model.

**Results:**

The global burden of NE has significantly declined over the past three decades. Between 1990 and 2021, the global ASIR decreased from 20.22 to 17.16, while the number of cases fell from 1,295,891.1 to 1,061,448.15, reflecting an 18.09% reduction. Similarly, the global ASMR declined from 13.81 in 1990 to 9.75 in 2021, with deaths decreasing from 883,082.06 to 603,605.62, representing a 31.65% reduction. Projections based on ARIMA models indicate that from 2022 to 2030, the global ASIR is expected to decline from 17.06 to 16.36, and the ASMR is projected to decrease from 9.55 to 7.98, suggesting a continued reduction in the burden of NE.

**Conclusion:**

This study illustrates the global progress made in reducing neonatal encephalopathy from 1990 to 2021; however, significant disparities persist. Targeted interventions are crucial to address these inequities and to achieve Sustainable Development Goal 3.2.

## 1 Introduction

Newborn mortality rates are crucial indicators of a country or region’s overall health and well-being (1, 2). Neonatal encephalopathy (NE), a condition that impacts 2 to 8 infants per 1,000 live births worldwide, poses a significant public health issue due to its considerable role in infant mortality and the risk of severe long-term neurodevelopmental impairments (3). As the third most common cause of death in children under the age of five, NE has a substantial effect on global morbidity and mortality figures, with about 1.4 million infants impacted each year (4). In clinical terms, NE is primarily characterized by changes in the level or quality of consciousness, often accompanied by seizures, dysfunction in cardiorespiratory systems, and atypical reflex responses (5).

Therapeutic hypothermia (TH) continues to be the sole intervention grounded in evidence for NE (6). However, its effectiveness seems largely confined to high-income nations, which represent only 10% of the overall NE incidence globally (7). The recent HELIX trial, recognized as the largest TH investigation undertaken in low- and middle-income countries (LMIC), has sparked serious concerns about the efficacy of TH in these environments (8). In contrast to initial expectations, the trial revealed no notable decrease in the combined primary outcome of mortality or neurodisability at the age of 2 years; rather, it was unexpectedly linked to higher mortality rates (8).

Concerning Sustainable Development Goal (SDG) 3.2, which seeks to reduce the global neonatal mortality rate (NMR) to below 12 deaths per 1,000 live births by the year 2030, there is a pressing need for innovative therapeutic strategies and enhanced healthcare interventions (9). Nonetheless, in spite of this urgent demand, a significant knowledge gap persists regarding the comprehensive comprehension of epidemiological trends associated with neonatal encephalopathy (NE) and their relationships with socioeconomic factors in different geographical settings. As a result, this study presents a detailed and systematic analysis of the burden of NE at the global, regional, and national levels, using the latest Global Burden of Disease (GBD) 2021 dataset. Our research greatly contributes to the current literature by providing thorough examinations of temporal trends and geographic variations in age-standardized incidence rates (ASIR) and age-standardized mortality rates (ASMR) attributable to NE. Moreover, our study not only highlights the varied distribution of the NE burden but also offers crucial evidence for developing targeted public health strategies and localized intervention plans to address this ongoing global health challenge.

## 2 Methods

### 2.1 Data source and framework

The results of GBD 2021 formed the basis for this research (available at https://vizhub.healthdata.org/gbd-results/), recognized as the most comprehensive and current evaluation of the impacts arising from 371 diseases and injuries at global, regional, and national levels (10). The analysis conducted in GBD 2021 included assessing the effects of these conditions and injuries, incorporating the calculation of a range of indicators (11). This research was bolstered by an extensive database comprising 100,983 sources (11). From this extensive dataset, 56,604 sources were leveraged, which included essential registration data, insights from verbal autopsies, and information gathered from surveys, population censuses, disease tracking systems, cancer registries, along with many other data types (12).

### 2.2 Definition of NE

Neonatal encephalopathy (NE) is a clinically defined syndrome of impaired neurological function in infants ≥35 weeks’ gestation, presenting within the first days of life with features such as altered consciousness, seizures, respiratory depression, and hypotonia (13). While the Global Burden of Disease (GBD) 2021 classifies NE under birth asphyxia/trauma (11), the etiology is heterogeneous and may include infections, metabolic disorders, or genetic causes (14, 15). This study adheres to the GBD framework, primarily reflecting hypoxic–ischemic encephalopathy (HIE).

### 2.3 Measures of disease burden

In this research, we collected annual data on the incidence and mortality related to neonatal encephalopathy spanning from 1990 to 2021. Detailed methodologies and definitions for these metrics were comprehensively explained in an earlier publication (11). Furthermore, the estimated annual percentage change (EAPC) acts as a crucial metric for assessing trends associated with these shifts (10). EAPC has demonstrated its efficacy in quantifying tendencies in age-standardized rates in the field of public health (16). All rates were reported per 100,000 individuals and presented along with a 95% uncertainty interval (UI), representing the 25th and 975th ordered values derived from 1,000 samples of the posterior distribution (17). The study adhered strictly to the standards outlined in the Guidelines for Accurate and Transparent Health Estimates Reporting (GATHER) (18).

### 2.4 Trend prediction

The prediction of age-standardized incidence rates (ASIR) and age-standardized mortality rates (ASMR) for the period spanning 2022 to 2030 was achieved through the use of an autoregressive integrated moving average (ARIMA) model. The subsequent methodology was adopted: the ARIMA model, which is tailored for time series forecasting, integrates autoregressive (AR) elements, moving average (MA) components, and differencing (d) to ensure the stability of the dataset (19). In the ARIMA (p, d, q) configuration, ‘p’ represents the number of autoregressive terms, ‘d’ denotes the degree of differencing applied, and ‘q’ signifies the count of moving average elements. To determine the optimal ARIMA(p, d, q) setup for forecasting trends in disease incidence and mortality rates, R software was employed. All visual interpretations and graphical displays were conducted using R statistical software (version 4.3.3).

### 2.5 Sociodemographic index

The Sociodemographic Index (SDI) serves as a composite measure that considers a country’s per capita income over time, average years of education, and the fertility rate among women under 25 (20). This indicator is more effective in representing both the burden of disease and the extent of healthy development.

## 3 Results

### 3.1 The incidence and changes of neonatal encephalopathy

Over the past three decades, the global burden of NE has significantly declined. The ASIR per 100,000 live births decreased from 20.22 (95% UI: 19.92–20.51) in 1990 to 17.16 (95% UI: 16.94–17.41) in 2021, representing an EAPC of −0.56 (95% CI: −0.62, −0.51). Concurrently, the number of cases fell by 18.09% (95% CI: −20.08, −16.10), from 1,295,891.1 (95% UI: 1,276,552.88–1,314,238.82) in 1990 to 1,061,448.15 (95% UI: 1,047,815.17–1,076,766.11) in 2021. However, this overall reduction masks substantial disparities. The burden remains markedly higher in low SDI regions, particularly Sub-Saharan Africa, where the 2021 ASIR was 27.43 (95% UI: 27.03–27.90)—nearly four times greater than that in high SDI regions (7.18; 95% UI: 7.02–7.33). Furthermore, male neonates consistently experienced a higher incidence globally, with an ASIR of 19.93 versus 14.18 for females in 2021.

The regional burden of NE varies significantly, with the highest ASIR observed in Sub-Saharan Africa and South Asia. In Eastern Sub-Saharan Africa, the ASIR decreased from 47.81 (95% UI: 46.74–48.98) in 1990 to 34.57 (95% UI: 33.84–35.28) in 2021, reflecting an EAPC of −1.06 (−1.21, −0.9), yet it remains the highest in the world. Similarly, Central Sub-Saharan Africa experienced a decline from 28.73 (95% UI: 27.51–30.04) to 21.27 (95% UI: 20.42–22.24), with an EAPC of −0.91 (−1.15, −0.67). In contrast, Southern Sub-Saharan Africa exhibited minimal change from 1990 to 2021, reporting an ASIR of 21.48 (95% UI: 21.02–21.96) in 2021, down from 22.26 (95% UI: 21.84–22.73) in 1990, indicating stagnation in this region. South Asia recorded an EAPC of −0.65 (−0.72, −0.58) in ASIR, decreasing from 18.14 (95% UI: 17.37–18.95) to 15.00 (95% UI: 14.38–15.69), thus remaining a high-burden area due to its large population and limited access to healthcare. The most significant decrease among all regions was noted in Southeast Asia, where the ASIR declined from 24.69 (95% UI: 23.95–25.35) to 14.14 (95% UI: 13.78–14.54), with an EAPC of −1.75 (−1.84, −1.66), likely attributable to targeted public health interventions. In contrast, high-income regions such as Western Europe and Australasia demonstrated generally low ASIRs during this period. In Western Europe, the ASIR fell from 7.39 (95% UI: 7.28–7.51) to 6.25 (95% UI: 6.14–6.35), while Australasia recorded the lowest ASIR in 2021 at 5.26 (95% UI: 5.05–5.48), reflecting the efficacy of the advanced healthcare systems in these regions.

**Figure 1:**
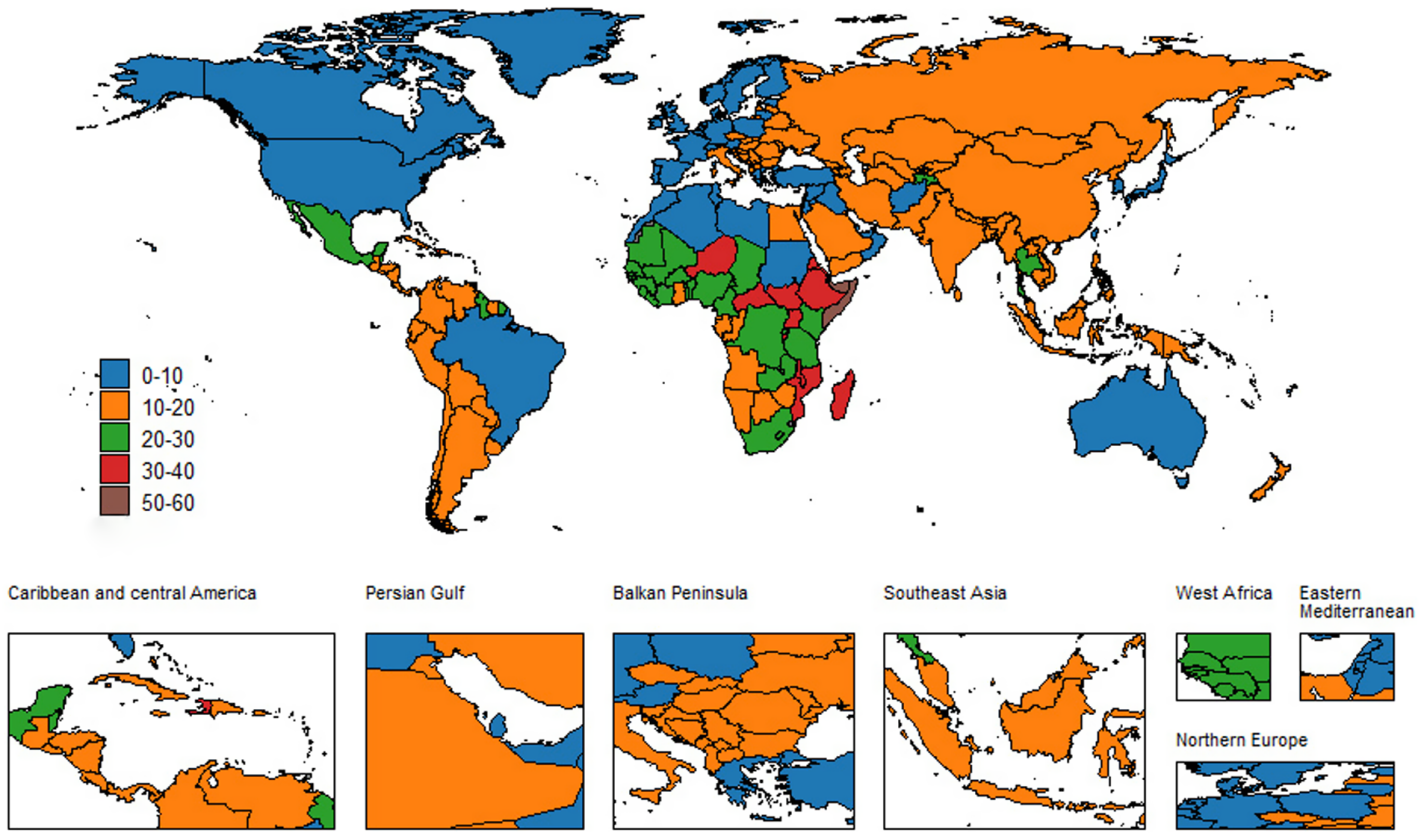
Global ASIR of neonatal encephalopathy in both sexes, 2021.

**Figure 2:**
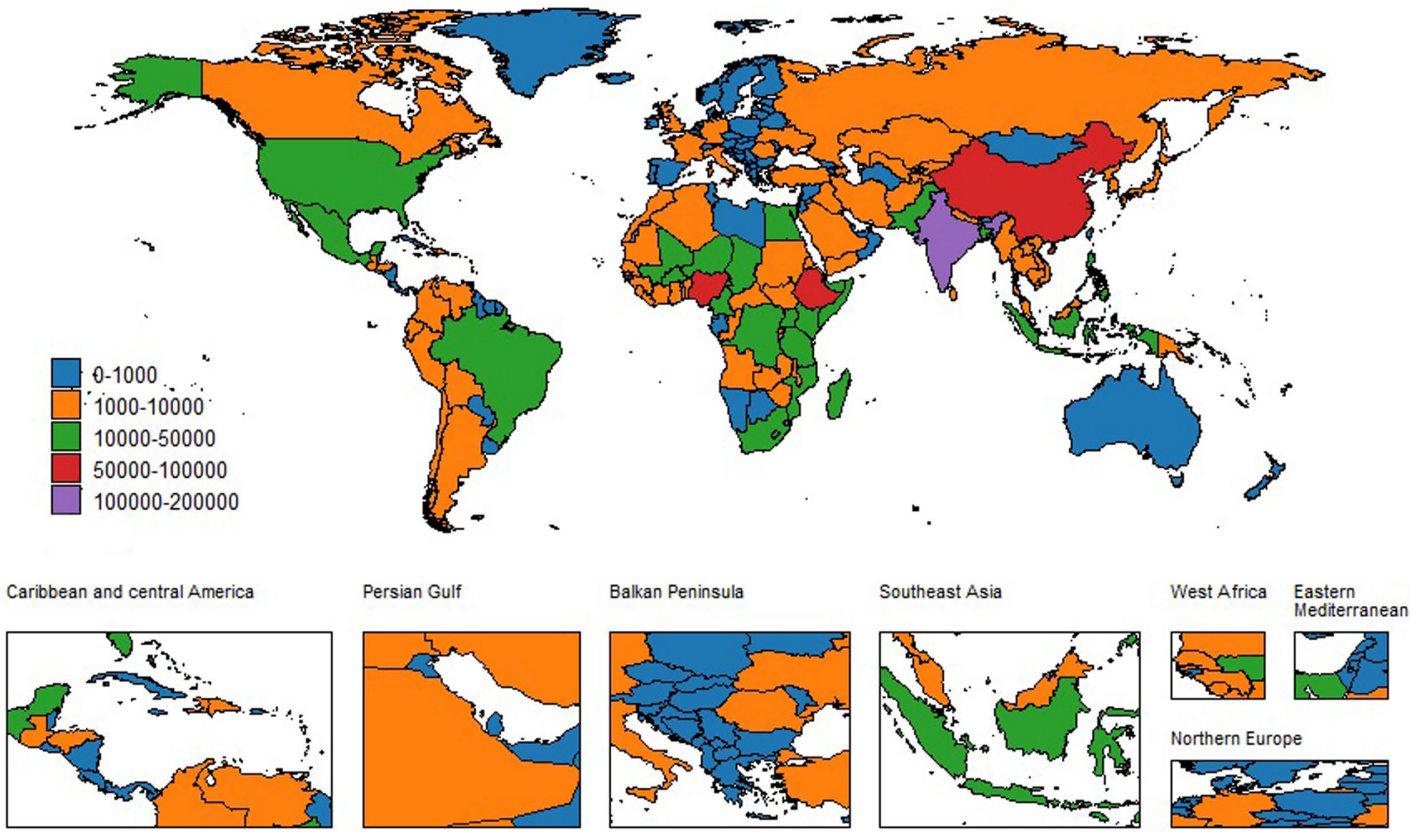
Global incident cases of neonatal encephalopathy in both sexes, 2021.

At the country level, disparities in the NE burden are even more pronounced. India and Nigeria continue to bear a high NE burden, while Belgium reports the lowest ASIR. Somalia’s ASIR has remained relatively stable at 56.13 (95% UI: 52.71–59.44) in 2021, compared to previous years. In stark contrast, Cambodia has shown a significant improvement, with an EAPC of −2.5 (−2.64,-2.35). India’s EAPC was −0.51 (−0.61, −0.41), with an ASIR of 16.11 (95% UI: 15.24–17.05) in 2021. Nigeria also exhibited a decline from 27.08 (95% UI: 25.5–28.71) to 22.87 (95% UI: 21.5–24.29), recording an EAPC of −0.57 (−0.65, −0.49). On the other hand, countries like Andorra and San Marino have consistently low ASIR; Andorra’s ASIR decreased from 7.41 (95% UI: 7.02–7.86) to 6.83 (95% UI: 6.43–7.19), while San Marino’s ASIR remained stable at 5.72 (95% UI: 5.40–6.08) in 2021. Afghanistan’s significant EAPC of −2.05 (−2.26, −1.84) in ASIR serves as an exemplary model for effective public health interventions in resource-poor settings. Other countries, such as Bhutan and Timor-Leste, showed promising improvements. Bhutan’s ASIR decreased from 17.51 (95% UI: 16.61–18.47) to 10.37 (95% UI: 9.79–10.98), with an EAPC of −1.81 (−1.89, −1.74), and Timor-Leste’s ASIR decreased from 41.47 (95% UI: 39.23–43.99) to 20.18 (95% UI: 19.02–21.34), with an EAPC of −2.75 (−2.94, −2.55). For detailed data, see Additional files 1–4 and Figures 1, 2.

### 3.2 The mortality and changes of neonatal encephalopathy

**Figure 3:**
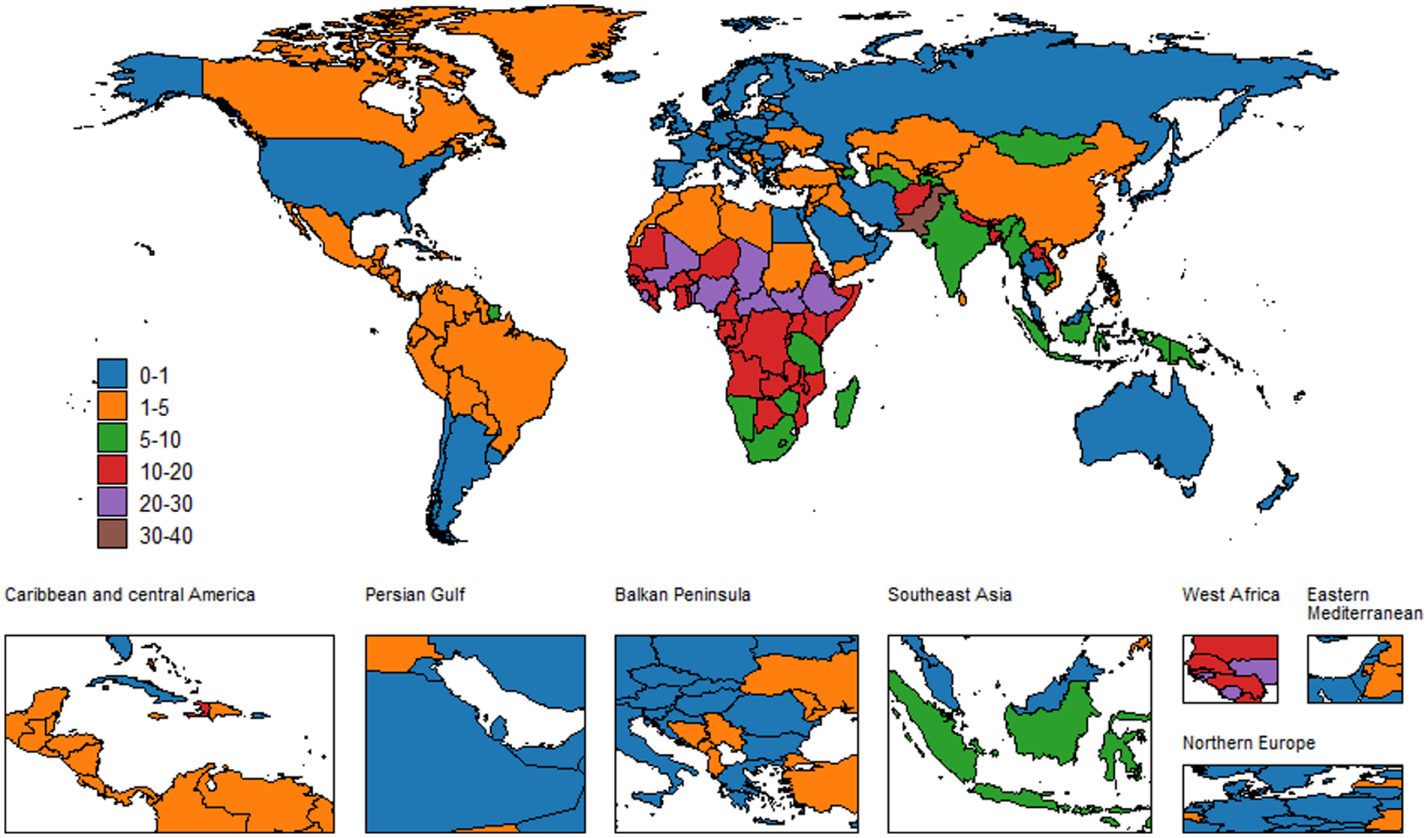
Global ASMR of neonatal encephalopathy in both sexes, 2021.

**Figure 4:**
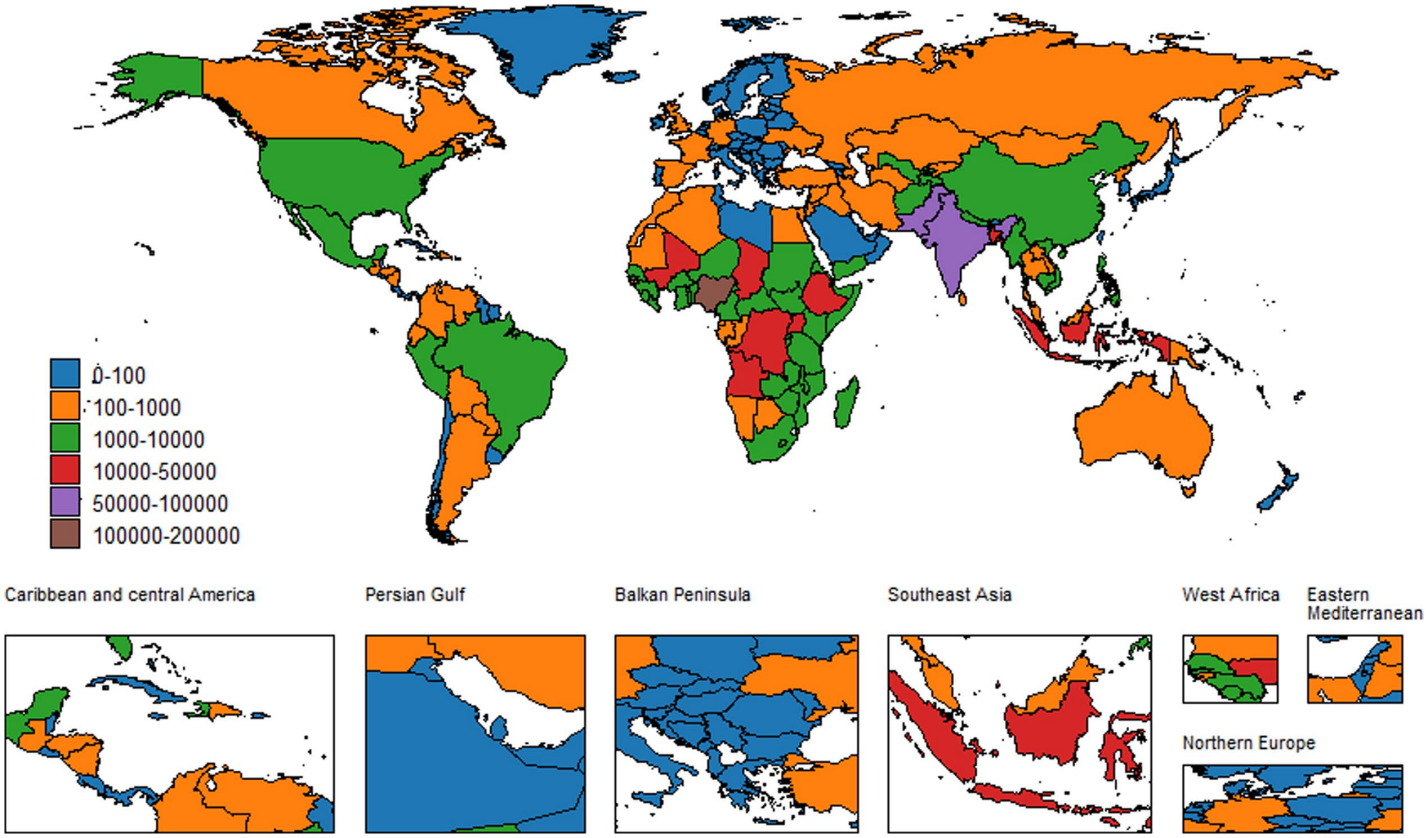
Global deaths from neonatal encephalopathy in both sexes, 2021.

**Figure 5:**
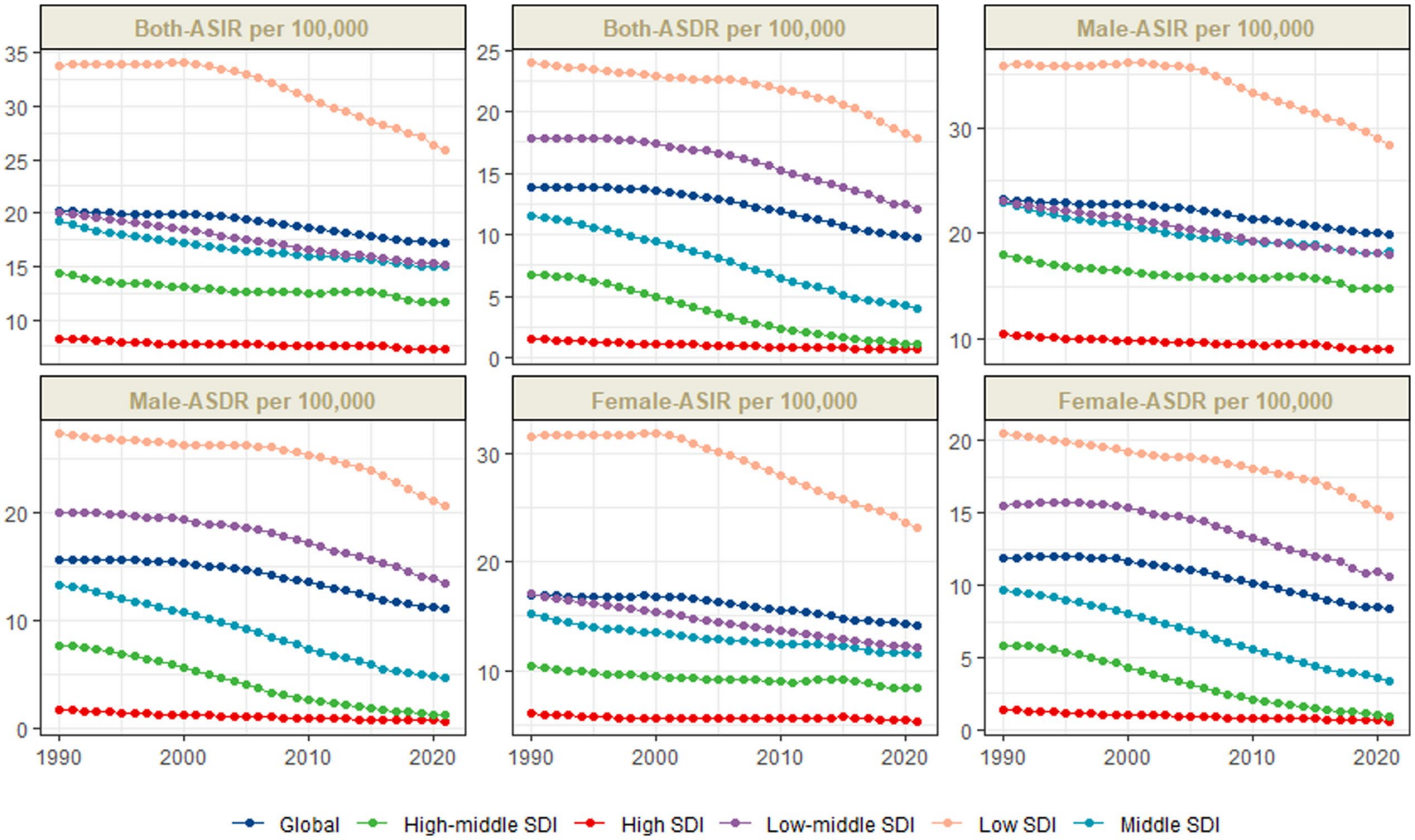
Trends in ASIR and ASMR of neonatal encephalopathy, 1990–2021.

Over the last 30 years, the global burden of mortality has decreased significantly. In 1990, the ASMR was 13.81 (95% UI: 12.65–15.71). By 2021, it fell to 9.75 (95% UI: 8.26–11.71), with an EAPC of −1.23 (−1.36, −1.11). Meanwhile, the annual number of deaths decreased from 8,830,82.06 (95% UI: 8,090,80.14–10,040,97.14) to 603,605.62 (95% UI: 511,190.63–725,270.82), which is a 31.65% decrease. However, large disparities by region and income remain. The greatest burden is observed in low SDI regions, particularly in Sub-Saharan Africa. Their ASMR was 23.94 (95% UI: 21.02–28.73) in 1990 and declined to 17.77 (95% UI: 14.77–21.58) in 2021, with an EAPC of −0.79 (−0.91, −0.68). In contrast, high SDI regions had the lowest ASMR, which decreased from 1.60 (95% UI: 1.51–1.71) in 1990 to 0.66 (95% UI: 0.58–0.72) in 2021, with an EAPC of −2.58 (−2.68, −2.48). Gender differences are also clear. Males have higher ASMRs than females worldwide. The male ASMR dropped from 15.63 (95% UI: 13.97–17.96) in 1990 to 11.07 (95% UI: 9.15–13.21) in 2021, with an EAPC of −1.20 (−1.33, −1.07). The female ASMR fell from 11.86 (95% UI: 10.52–13.70) to 8.34 (95% UI: 6.95–9.94), with an EAPC of −1.28 (−1.41, −1.15) (Figures 3–5).

Countries with a high burden include Pakistan and the Central African Republic. Significant reductions were observed in Afghanistan, where ASMR decreased from 29.12 (95% UI: 21.24–37.08) in 1990 to 15.65 (95% UI: 11.66–20.00) in 2021, yielding an EAPC of −1.74 (−1.82, −1.65). Angola also demonstrated improvement, with its ASMR falling from 32.70 (95% UI: 23.98–42.30) to 17.35 (95% UI: 12.67–22.25), resulting in an EAPC of −2.04 (−2.20, −1.89). In contrast, Andorra and San Marino maintained extremely low ASMR. Andorra’s ASMR declined from 1.10 (95% UI: 0.76–1.49) to 0.14 (95% UI: 0.07–0.21), with an EAPC of −4.87 (−5.33, −4.41). Similarly, the ASMR in San Marino decreased from 3.16 (95% UI: 2.33–4.11) to 0.49 (95% UI: 0.30–0.77), accompanied by an EAPC of −5.11 (−5.36, −4.85).

It is highly variable in terms of the mortality burden among different regions. The ASMR were highest in sub-Saharan Africa and South Asia. In Eastern Sub-Saharan Africa, the ASMR went down from 23.37 in 1990 to 15.39 in 2021, with a 95% UI of 19.98–28.27 and 12.33–19.21, respectively, while the EAPC was −1.27 (−1.45, −1.09), but remains one of the highest across the world. In Central Sub-Saharan Africa, this went down from 25.04 (95% UI: 19.08–31.43) to 17.66 (95% UI: 13.95–22.43), while the EAPC was −0.67 (−0.92, −0.42). Southern Sub-Saharan Africa showed a very minimal decline in its ASMR, which fell from 11.92 (95% UI: 10.07–14.20) in 1990 to 8.28 (95% UI: 6.39–10.82) in 2021, at an EAPC of −1.15 (−1.29, −1.02).

Despite an EAPC of −1.25 (−1.36, −1.14), South Asia still had a high burden. The large population, combined with limited healthcare access, means that its ASMR has only decreased from 18.95 (95% UI: 15.74–25.06) in 1990 to 12.94 (95% UI: 10.23–16.84) in 2021. Meanwhile, Southeast Asia saw a remarkable decline: from 8.94 (95% UI: 6.66–10.94) to 4.52 (95% UI: 3.21–5.63), its EAPC reaching −2.20 (−2.34, −2.06). This is likely the result of targeted public health efforts. High-income regions, including Western Europe and Australasia, maintained very low ASMR globally. In Western Europe, the ASMR decreased from 1.47 (95% UI: 1.43–1.52) to 0.65 (95% UI: 0.56–0.74), while in Australasia, it declined from 1.27 (95% UI: 1.18–1.37) in 1990 to 0.75 (95% UI: 0.62–0.91) in 2021, with an EAPC of −1.01 (−1.61, −0.41). These trends indicate the effectiveness of advanced healthcare systems.

These differences are even more significant at the country level. At the country level, India’s ASMR fell from 15.08 (95% UI: 11.79–21.71) in 1990 to 7.40 (95% UI: 5.12–12.09) in 2021, with an EAPC of −2.32 (−2.46, −2.19). Nigeria’s ASMR fell from 33.55 (95% UI: 28.22–43.61) to 25.40 (95% UI: 19.95–31.38), with an EAPC of −0.73 (−0.84, −0.61). Andorra and San Marino continued to have very low ASMR. The ASMR declined from 1.10 (95% UI: 0.76–1.49) to 0.14 (95% UI: 0.07–0.21) in Andorra, with an EAPC of −4.87 (−5.33, −4.41); from 3.16 (95% UI: 2.33–4.11) to 0.49 (95% UI: 0.30–0.77) in San Marino, with an EAPC of −5.11 (−5.36, −4.85). Meanwhile, the best practice of a drop in NMR in cases such as that observed in Afghanistan is represented by −1.74 (−1.82, −1.65) due to effective public health efforts made in low-resource areas. In contrast, Somalia’s ASMR remained nearly unchanged, from 19.73 (95% UI: 12.99–31.65) in 1990 to 19.99 (95% UI: 13.59–31.97) in 2021, reflecting little progress. Other countries that showed improvement included Bhutan and Timor-Leste. Bhutan’s ASMR declined from 26.94 (95% UI: 18.99–35.75) to 11.69 (95% UI: 7.73–16.27), with an EAPC of −2.97 (−3.20, −2.74). Timor-Leste had an ASMR that decreased from 13.89 (95% UI: 9.00–21.46) to 7.48 (95% UI: 4.42–11.28), with an EAPC of −2.14 (−2.20, −2.07). See Additional files 5–8 for all the above.

### 3.3 Burden of NE based on SDI

**Figure 6:**
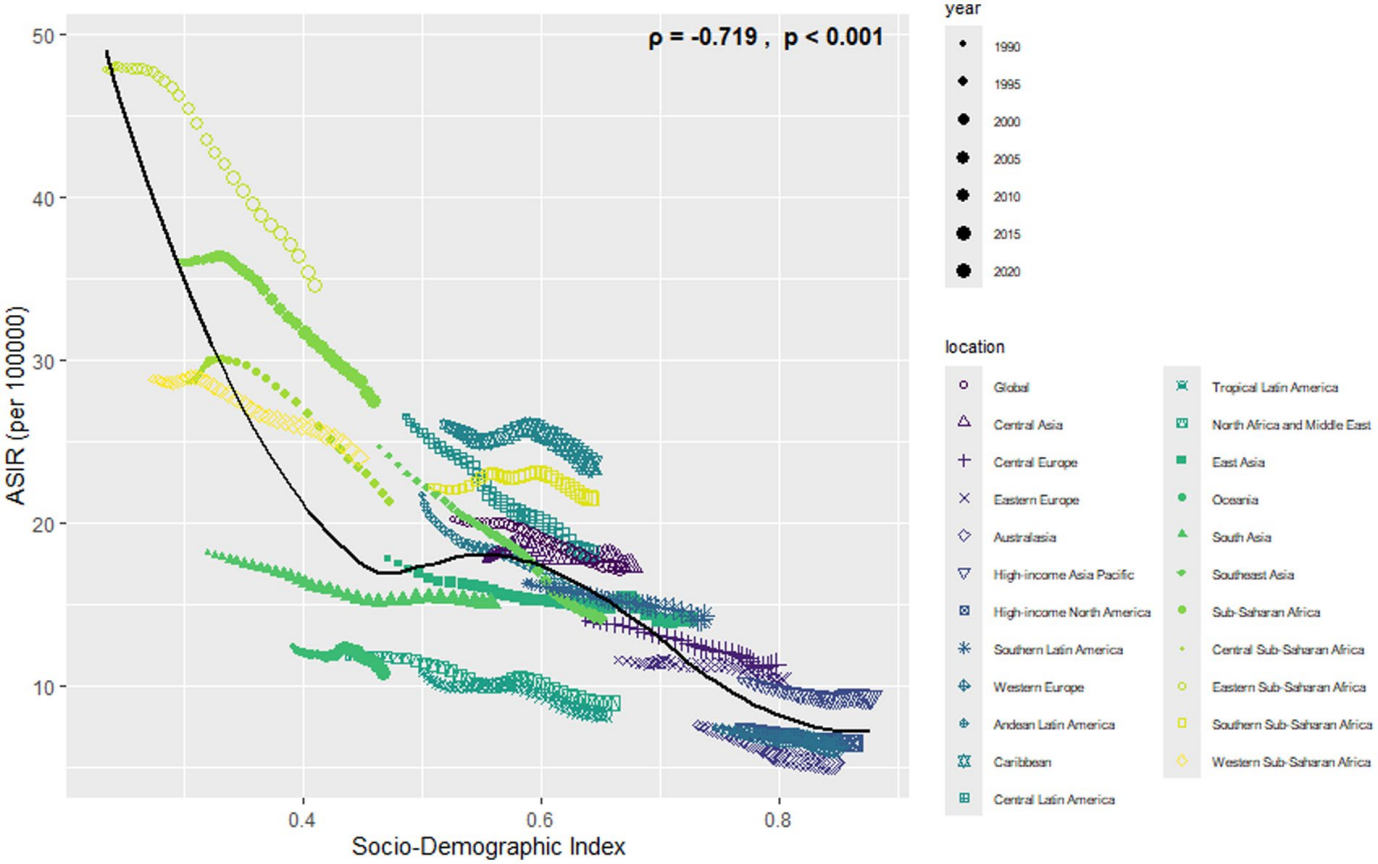
Association between ASIR of neonatal encephalopathy and regional SDI, 1990–2021.

**Figure 7:**
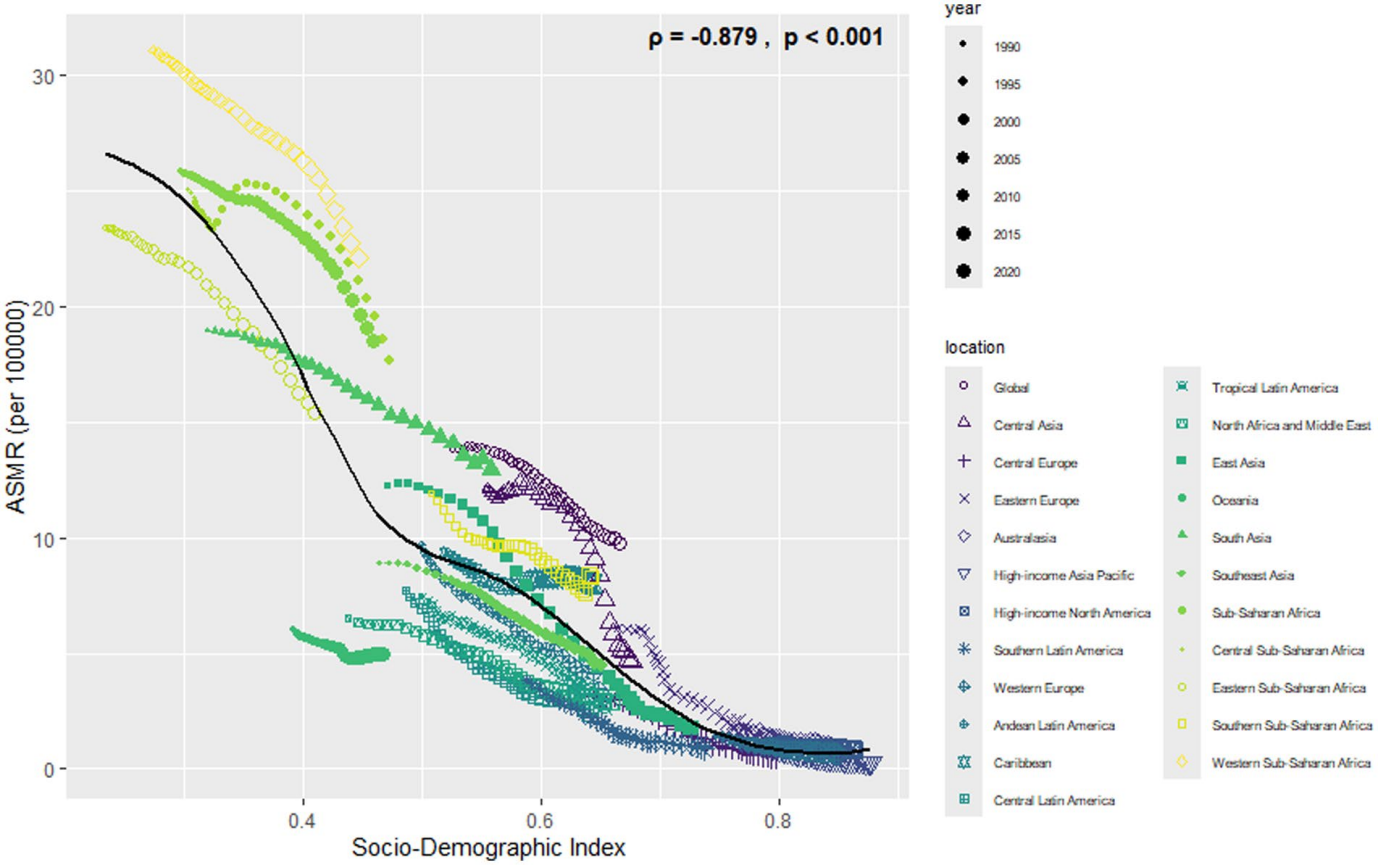
Association between ASMR of neonatal encephalopathy and regional SDI, 1990–2021.

Most cases and deaths linked to NE were found in regions with middle to lower-middle SDI levels and in low- and middle-income countries (see Additional files 3, 4, 7, 8). In all regions, there was a clear link showing that as SDI increases, ASIR and ASDR decrease (see Figures 6, 7).

### 3.4 Prediction of age-standardized rate by ARIMA models

**Figure 8:**
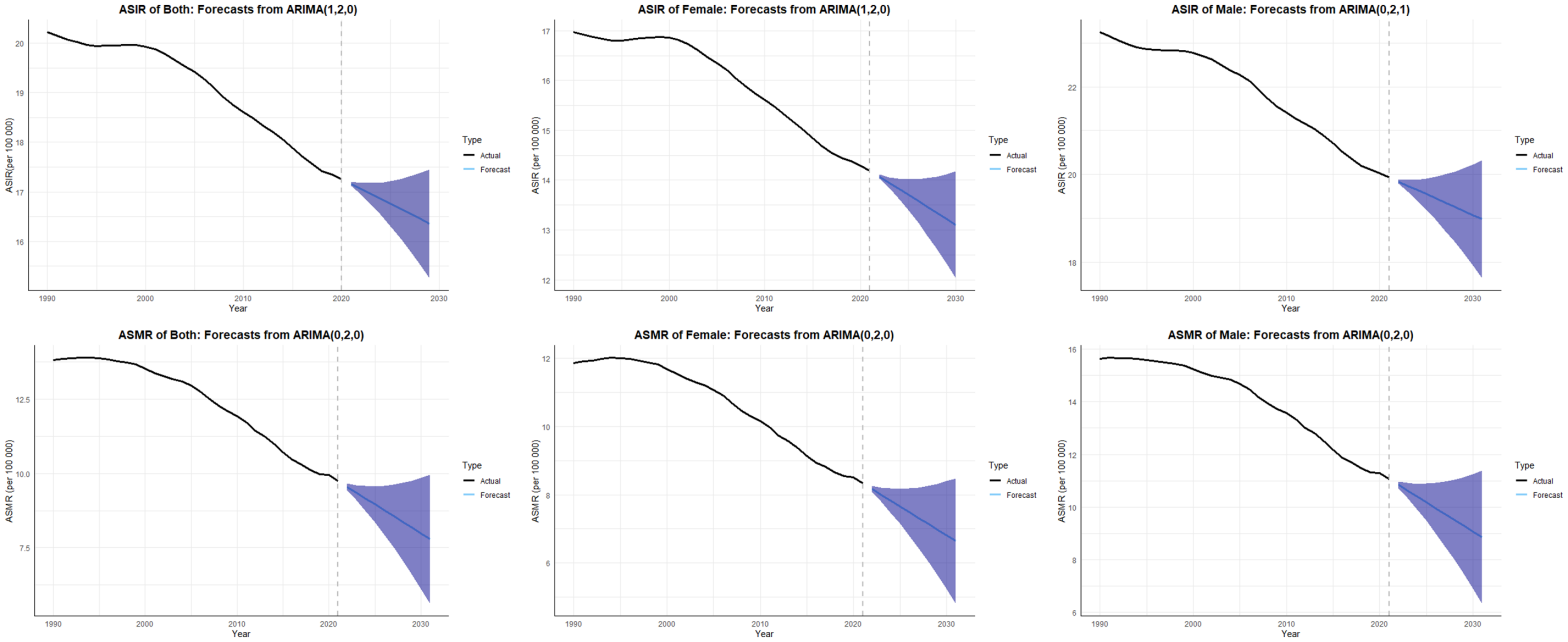
Projected ASIR and ASMR of neonatal encephalopathy from 2022 to 2030, by Sex.

The predicted results for ASIR and ASMR from 2022 to 2030 are shown in Figure 8. During this time, the ASIR for both sexes is expected to drop from 17.06 to 16.36 per 100,000. For males, the ASIR will likely fall from 19.84 to 19.08 per 100,000. For females, it is expected to decrease from 14.07 to 13.10 per 100,000. Similarly, the ASMR for both sexes is predicted to decline from 9.55 to 7.98 per 100,000. Males will see a drop from 10.85 to 9.08 per 100,000, and females will see a decrease from 8.17 to 6.81 per 100,000. From 2022 to 2030, the ASMR for both sexes is expected to fall from 9.55 to 7.98 per 100,000, showing a steady decline. For females, the ASMR will likely drop from 8.17 to 6.81 per 100,000. For males, it is predicted to decrease from 10.85 to 9.08 per 100,000. This data shows that mortality rates will continue to decrease for all genders.

## 4 Discussion

This analysis provides a comprehensive examination of the global, regional, and national burdens of NE from 1990 to 2021, utilizing data from the GBD 2021. The findings reveal notable reductions in both the ASIR and ASMR of NE over the past 30 years. Nevertheless, significant disparities remain across different regions and socioeconomic classes, particularly in LMIC, highlighting the need for targeted public health interventions (21, 22).

Regional differences in the burden of NE are particularly noteworthy. In 2021, very high ASIR were reported in Sub-Saharan Africa and South Asia, with figures of 34.57 and 15.00 per 100,000, respectively. In contrast, regions with higher income levels, such as Western Europe and Australasia, typically exhibit lower ASIR. Australasia reported an ASIR of 5.26 per 100,000 during the same year. These findings align with previous studies that highlight the impact of socioeconomic factors on neonatal health outcomes (4, 23). Nations characterized by high SDI scores consistently demonstrate low ASIR and ASMR, underscoring the effectiveness of their robust healthcare systems in reducing rates of neonatal morbidity and mortality (24).

Low and middle socioeconomic status (SES) has a significant adverse impact on neonatal outcomes. Research indicates that neonates from low-income families face higher risks in terms of health status and survival rates (25). For instance, one study found that compared to families with sufficient income, those with insufficient income had worse maternal assessments of overall infant health and higher infant hospitalization rates (26). Moreover, low SES is closely associated with adverse pregnancy outcomes such as preterm birth and low birth weight (27). These effects are particularly pronounced in low- and middle-income countries (LMIC), where healthcare resources are limited and social support systems are inadequate (28). Specifically, pregnant women with low SES often lack adequate prenatal care, which increases the risk of preterm birth and low birth weight (28). Additionally, these families may not have access to timely medical services, resulting in untreated neonatal illnesses (26). In regions such as sub-Saharan Africa and South Asia, where socioeconomic conditions are poor, neonatal mortality rates remain high (28). To improve this situation, targeted public health interventions are necessary. These measures include enhancing the education level of pregnant women, improving prenatal and postnatal care services, and providing economic support to mitigate the impact of poverty on neonatal health (27). Furthermore, strengthening community health services and increasing the accessibility of medical resources are also key (28). Through these measures, the adverse effects of socioeconomic inequality on neonatal outcomes can be effectively reduced, and the global neonatal health goals can be achieved (28).

Our research also reveals significant gender differences in the impact of NE. Male infants consistently exhibit higher ASIR and ASMR globally compared to female infants. In 2021, the ASIR for males was recorded at 19.93 per 100,000, whereas the figure for females was 14.18. Similarly, males had an ASMR of 11.07 per 100,000, in contrast to 8.34 for females. These findings are consistent with previous studies that suggest male neonates may be at a greater risk for adverse outcomes during the neonatal period (29).

The most significant finding in this study shows that the SDI has an inverse association with the burden of neonatal events. Low- and middle-SDI settings make significant contributions to overall incidence and mortality due to NE. It is true particularly in countries like India and Nigeria, which, despite remarkable declines in ASIR and ASMR over the past three decades, have continued to remain hotspots. For instance, in India, ASIR has been reduced from 20.22 in 1990 to 16.11 by 2021, while in Nigeria, the same rate declined from 27.08 to 22.87 between 1990 and 2021. They do, however, still contribute to an important percentage of the global burden of active NE cases, hence highlighting the pressing need for targeted interventions in these regions (24).

Clinical practices grounded in evidence have significantly improved the survival rates of newborns affected by NE (30, 31). For instance, recent findings from the Indian Neonatal Collaborative (INNC) revealed that among infants suffering from moderate or severe hypoxic–ischemic encephalopathy (HIE), there is an 82% survival rate at the time of discharge (32). Although fully eradicating neonatal mortality resulting from NE is a considerable challenge, even slight decreases can yield substantial economic advantages for countries and enhance human productivity. Achieving these reductions necessitates the establishment of research priorities, the creation of consensus-based guidelines at the community level, and the assurance that expectant mothers have access to care that is both affordable and available (33).

Perinatal asphyxia, complicated by conditions such as prolonged labor, pre-eclampsia, and antepartum hemorrhage, is one of the major causes of neonatal encephalopathy and stillbirths in developing countries (34). Over 50 million home deliveries annually do not have skilled birth attendants in LMIC; simultaneously, facility-based births experience high-quality intrapartum care with scant availability to neonatal intensive care (35). These challenges further stress the pressing need for targeted research into the aetiology and timing of neonatal encephalopathy, the evaluation of the role of effective intrapartum monitoring, and the development of strategies for providing essential neonatal supportive care.

In high-income countries, the incidence of moderate-to-severe HIE has plateaued at about 1 in 1000 births, with a slight decrease in the most severe cases and some reduction in overall mortality rates (36). Survivors of neonatal encephalopathy have lifelong challenges that include intellectual disabilities, cerebral palsy, hearing loss, and visual impairments (37). These conditions affect not only individuals and families but also result in significant indirect costs that often exceed direct medical costs. Thus, current trends in disabilities due to neonatal encephalopathy, preterm birth complications, and other neonatal conditions should be a priority in national newborn survival strategies. Integration of these insights into cost–benefit analyses can help optimize health financing and resource allocation.

Previous studies have established a clear link between health expenditure and under-five mortality (38, 39). In developed countries, higher public spending on health yields expected outcomes, as evidenced by low infant mortality rates (40). In developing regions, even moderate increases in government spending on health can lead to significant improvements in newborn survival, although some disparities may persist (41). Beyond health expenditure, social and environmental enhancements are essential for reducing health inequities in LMIC, which are often driven by increased non-health public investments. Additionally, factors such as poverty, maternal education, urbanization, and the accessibility of quality prenatal care, skilled birth attendance, and maternal vaccinations significantly influence neonatal survival rates (42).

While ARIMA models predict a continued decline in NE burden from 2022 to 2030, these projections should be interpreted cautiously. The model assumes linear continuation of past trends, and does not account for emerging non-asphyxial etiologies, variable implementation of therapeutic hypothermia in LMICs, or disruptions in healthcare systems due to conflicts, pandemics, or policy changes. Thus, the generalizability of these forecasts is limited, and they should be used as scenario-based estimates rather than definitive policy targets.

This study has key limitations. Despite GBD 2021 providing comprehensive global estimates, data quality varies significantly by national income. Underreporting of neonatal deaths and cause misclassification, prevalent in low-income countries due to incomplete vital registration and limited diagnostic capacity (especially in Sub-Saharan Africa and South Asia) (43, 44), likely underestimate the true burden of NE. Inconsistent definitions (e.g., birth asphyxia, hypoxic–ischemic encephalopathy, NE) further compromise data accuracy. While GBD methodology partially mitigates this through etiology reallocation and omission of unrelated ICD codes, results in resource-limited settings demand cautious interpretation. Additional constraints include: data accessibility compromised by reporting delays, conflict, natural disasters, or governance disruptions; variable mortality documentation quality; and the GBD assumption of a single underlying cause, which may not reflect clinical complexities involving multiple contributing factors. Moreover, primary data inaccuracies persist, particularly in lower-income nations with weak surveillance systems.

## 5 Conclusion

This study provides a comprehensive analysis of the global, regional, and national impacts of NE from 1990 to 2021. While significant progress has been made in reducing the global burden of NE, persistent disparities remain across different regions and economic groups. Targeted public health interventions are urgently needed to address these inequities. The findings offer valuable insights for policymakers and healthcare providers aiming to improve neonatal health outcomes and achieve SDG 3.2, which seeks to reduce neonatal mortality to fewer than 12 deaths per 1,000 live births by 2030. This research underscores the importance of optimizing resource allocation, guiding public health planning, and implementing targeted strategies to enhance the prevention and management of NE on a global scale.

## Data Availability

All datasets analysed in this study are publicly accessible through the Global Burden of Disease Results portal at https://vizhub.healthdata.org/gbd-results/.
