# Supplementary material for: Analysis of global, regional, and national burdens of neonatal encephalopathy from 1990 to 2021: insights from the Global Burden of Disease Study 2021
Source: Front Public Health. 2025 Oct 8;13:1627448. doi: 10.3389/fpubh.2025.1627448 (PMC12540312; doi:10.3389/fpubh.2025.1627448)
Supplement: Supplementary file 1 [file Table_1.doc]

| Location | Incidence | | |
| --- | --- | --- | --- |
|  | ASIR (95% UI) | | EAPC  (95% CI) |
|  | 1990 | 2021 |  |
| Global | 20.22 (19.92,20.51) | 17.16(16.94, 17.41) | -0.56(-0.62, -0.51) |
| Sex | | | |
| Female | 16.97 (16.56,17.43) | 14.18(13.88, 14.51) | -0.63(-0.70, -0.56) |
| Male | 23.25 (22.89,23.66) | 19.93(19.67, 20.24) | -0.52(-0.56, -0.47) |
| Low SDI | 33.85 (33.33,34.41) | 25.84 (25.47,26.22) | -0.87 (-1,-0.75) |
| Low-middle SDI | 20.12 (19.64,20.61) | 15.14 (14.82,15.45) | -0.95 (-0.97,-0.93) |
| Middle SDI | 19.25 (18.81,19.68) | 15.05 (14.77,15.33) | -0.75 (-0.79,-0.71) |
| High-middle SDI | 14.35 (13.88,14.8) | 11.72 (11.42,12.04) | -0.53 (-0.59,-0.47) |
| High SDI | 8.27 (8.11,8.45) | 7.18 (7.02,7.33) | -0.35 (-0.39,-0.31) |
| Central Asia | 17.73 (17.29,18.15) | 17.35 (16.89,17.83) | -0.15 (-0.21,-0.08) |
| Central Europe | 13.96 (13.72,14.21) | 11.28 (11.09,11.48) | -0.8 (-0.83,-0.77) |
| Eastern Europe | 11.59 (11.16,12.02) | 10.32 (9.91,10.77) | -0.36 (-0.43,-0.29) |
| High-income | 8.65 (8.49,8.82) | 7.24 (7.1,7.37) | -0.53 (-0.56,-0.51) |
| Australasia | 7.56 (7.25,7.88) | 5.26 (5.05,5.48) | -1.29 (-1.39,-1.2) |
| High-income Asia Pacific | 10.37 (10.14,10.61) | 9.31 (9.18,9.45) | -0.29 (-0.36,-0.21) |
| High-income North America | 7.48 (7.09,7.88) | 6.47 (6.13,6.8) | -0.49 (-0.52,-0.47) |
| Southern Latin America | 16.31 (15.59,17) | 14.28 (13.74,14.88) | -0.43 (-0.46,-0.39) |
| Western Europe | 7.39 (7.28,7.51) | 6.25 (6.14,6.35) | -0.48 (-0.53,-0.43) |
| Andean Latin America | 21.67 (20.91,22.51) | 14.96 (14.41,15.53) | -1.09 (-1.15,-1.03) |
| Caribbean | 26.05 (25.32,26.83) | 23.49 (22.71,24.33) | -0.23 (-0.3,-0.16) |
| Central Latin America | 26.54 (26.24,26.88) | 18.12 (17.91,18.35) | -1.26 (-1.3,-1.22) |
| Tropical Latin America | 10.78 (10.59,10.97) | 8.26 (8.14,8.39) | -0.8 (-0.92,-0.68) |
| North Africa and Middle East | 11.8 (11.56,12.04) | 8.94 (8.76,9.13) | -0.96 (-1.05,-0.86) |
| East Asia | 17.78 (16.76,18.75) | 14.01 (13.25,14.75) | -0.63 (-0.71,-0.55) |
| Oceania | 12.34 (11.86,12.86) | 10.82 (10.32,11.36) | -0.21 (-0.31,-0.11) |
| South Asia | 18.14 (17.37,18.95) | 15 (14.38,15.69) | -0.65 (-0.72,-0.58) |
| Southeast Asia | 24.69 (23.95,25.35) | 14.14 (13.78,14.54) | -1.75 (-1.84,-1.66) |
| Sub-Saharan Africa | 36.03 (35.5,36.61) | 27.43 (27.03,27.9) | -0.9 (-1.03,-0.76) |
| Central Sub-Saharan Africa | 28.73 (27.51,30.04) | 21.27 (20.42,22.24) | -0.91 (-1.15,-0.67) |
| Eastern Sub-Saharan Africa | 47.81 (46.74,48.98) | 34.57 (33.84,35.28) | -1.06 (-1.21,-0.9) |
| Southern Sub-Saharan Africa | 22.26 (21.84,22.73) | 21.48 (21.02,21.96) | -0.06 (-0.14,0.02) |
| Western Sub-Saharan Africa | 28.81 (28.03,29.56) | 24.03 (23.35,24.7) | -0.58 (-0.65,-0.51) |
