# Supplementary material for: Analysis of global, regional, and national burdens of neonatal encephalopathy from 1990 to 2021: insights from the Global Burden of Disease Study 2021
Source: Front Public Health. 2025 Oct 8;13:1627448. doi: 10.3389/fpubh.2025.1627448 (PMC12540312; doi:10.3389/fpubh.2025.1627448)
Supplement: Supplementary file 2 [file Table_2.doc]

| Location | Incidence | | |
| --- | --- | --- | --- |
|  | ASIR (95% UI) | | EAPC  (95% CI) |
|  | 1990 | 2021 |  |
| Afghanistan | 16.55 (15.52,17.53) | 9.04 (8.5,9.57) | -2.05 (-2.26,-1.84) |
| Albania | 19.79 (18.61,20.82) | 13.96 (13.13,14.85) | -1.35 (-1.45,-1.25) |
| Algeria | 7.28 (6.82,7.73) | 6.02 (5.67,6.39) | -0.86 (-0.95,-0.76) |
| American Samoa | 6.77 (6.4,7.16) | 8.93 (8.5,9.48) | 1.16 (1.03,1.29) |
| Andorra | 7.41 (7.02,7.86) | 6.83 (6.43,7.19) | 0.11 (-0.09,0.3) |
| Angola | 30.27 (28.44,32.04) | 16.78 (15.83,17.82) | -2.06 (-2.16,-1.96) |
| Antigua and Barbuda | 16.55 (15.66,17.5) | 15.77 (14.94,16.63) | -0.1 (-0.16,-0.04) |
| Argentina | 15.2 (14.34,16.11) | 13.93 (13.21,14.78) | -0.2 (-0.27,-0.14) |
| Armenia | 17.84 (16.95,18.82) | 17.8 (16.85,18.8) | 0.04 (-0.05,0.13) |
| Australia | 5.91 (5.57,6.27) | 3.93 (3.71,4.18) | -1.45 (-1.64,-1.26) |
| Austria | 9.39 (8.88,9.94) | 8.05 (7.6,8.53) | -0.57 (-0.66,-0.47) |
| Azerbaijan | 17.67 (16.73,18.66) | 16.3 (15.46,17.23) | -0.37 (-0.49,-0.26) |
| Bahamas | 15.02 (14.24,15.93) | 14.38 (13.66,15.14) | -0.06 (-0.12,0) |
| Bahrain | 12.44 (11.72,13.18) | 8.73 (8.22,9.25) | -1.03 (-1.14,-0.92) |
| Bangladesh | 19.66 (18.62,20.99) | 12.54 (11.86,13.29) | -1.28 (-1.36,-1.2) |
| Barbados | 16.46 (15.62,17.43) | 15.84 (14.99,16.73) | -0.05 (-0.08,-0.02) |
| Belarus | 12.33 (11.67,12.97) | 10.69 (10.15,11.33) | -0.42 (-0.52,-0.33) |
| Belgium | 4.58 (4.32,4.84) | 3.37 (3.19,3.56) | -1.05 (-1.15,-0.96) |
| Belize | 27.93 (26.42,29.59) | 23.26 (22.03,24.67) | -0.63 (-0.7,-0.56) |
| Benin | 30.13 (28.49,32.1) | 22.25 (21.02,23.62) | -0.88 (-0.94,-0.82) |
| Bermuda | 12.97 (12.29,13.69) | 11.08 (10.46,11.76) | -0.47 (-0.51,-0.43) |
| Bhutan | 17.51 (16.61,18.47) | 10.37 (9.79,10.98) | -1.81 (-1.89,-1.74) |
| Bolivia (Plurinational State of) | 25.42 (23.94,27) | 16.62 (15.69,17.64) | -1.28 (-1.35,-1.21) |
| Bosnia and Herzegovina | 22.17 (20.89,23.36) | 17.26 (16.33,18.27) | -1.1 (-1.21,-1) |
| Botswana | 18.29 (17.26,19.46) | 14.9 (14.04,15.92) | -0.65 (-0.69,-0.62) |
| Brazil | 10.53 (10.33,10.73) | 8.12 (8,8.25) | -0.78 (-0.9,-0.66) |
| Brunei Darussalam | 8.11 (7.7,8.65) | 7.53 (7.14,7.93) | -0.17 (-0.22,-0.11) |
| Bulgaria | 17.68 (16.73,18.71) | 15.99 (15.14,16.87) | -0.33 (-0.43,-0.22) |
| Burkina Faso | 34.42 (32.39,36.51) | 27.19 (25.64,28.9) | -0.83 (-0.93,-0.73) |
| Burundi | 44.96 (42.54,47.95) | 39.57 (37.22,41.84) | -0.51 (-0.68,-0.35) |
| Cabo Verde | 23.39 (22,24.94) | 19.42 (18.31,20.55) | -0.72 (-0.94,-0.5) |
| Cambodia | 38.6 (36.38,40.96) | 19.36 (18.35,20.57) | -2.5 (-2.64,-2.35) |
| Cameroon | 22.78 (21.48,24.15) | 20.83 (19.6,22.07) | -0.22 (-0.3,-0.13) |
| Canada | 6.57 (6.2,6.96) | 5.76 (5.45,6.06) | -0.43 (-0.47,-0.39) |
| Central African Republic | 33.45 (31.54,35.72) | 30.02 (28.35,31.97) | -0.25 (-0.33,-0.16) |
| Chad | 35 (32.77,37.3) | 28.48 (26.88,30.27) | -0.67 (-0.81,-0.53) |
| Chile | 19.62 (18.54,20.74) | 15.79 (14.86,16.64) | -0.86 (-0.96,-0.76) |
| China | 17.87 (16.8,18.88) | 14 (13.22,14.77) | -0.65 (-0.73,-0.57) |
| Colombia | 15.33 (14.53,16.19) | 12.13 (11.43,12.91) | -0.73 (-0.8,-0.66) |
| Comoros | 40.96 (38.39,43.76) | 27.25 (25.7,29.11) | -1.3 (-1.34,-1.26) |
| Congo | 23.82 (22.45,25.21) | 18.5 (17.45,19.73) | -0.85 (-0.91,-0.79) |
| Cook Islands | 5.87 (5.54,6.17) | 6.13 (5.8,6.43) | 0.35 (0.28,0.43) |
| Costa Rica | 12.58 (11.87,13.32) | 11.06 (10.42,11.7) | -0.31 (-0.36,-0.26) |
| Côte d'Ivoire | 27.41(25.86,29.27) | 21.42(20.26,22.68) | -0.66(-0.77, -0.56) |
| Croatia | 17.09 (16.3,18) | 14.87 (14.05,15.65) | -0.52 (-0.56,-0.49) |
| Cuba | 15.49 (14.69,16.35) | 13.98 (13.19,14.75) | -0.25 (-0.34,-0.16) |
| Cyprus | 7.54 (7.14,7.99) | 5.71 (5.39,6.06) | -0.99 (-1.03,-0.94) |
| Czechia | 19.49 (18.45,20.67) | 14.8 (14,15.64) | -0.72 (-0.85,-0.59) |
| Democratic People's Republic of Korea | 18.41 (17.35,19.49) | 16.91 (15.93,17.9) | -0.25 (-0.34,-0.16) |
| Democratic Republic of the Congo | 28.35 (26.64,30.14) | 22.81 (21.5,24.32) | -0.57 (-0.88,-0.26) |
| Denmark | 8.48 (8.02,8.96) | 7.15 (6.78,7.57) | -0.54 (-0.67,-0.42) |
| Djibouti | 24.85 (23.37,26.3) | 22.54 (21.35,23.91) | -0.23 (-0.31,-0.15) |
| Dominica | 19.14 (18.19,20.25) | 16.34 (15.44,17.25) | -0.41 (-0.47,-0.35) |
| Dominican Republic | 21.75 (20.62,23) | 17.07 (16.19,17.99) | -0.6 (-0.67,-0.52) |
| Ecuador | 18.02 (17.06,19.07) | 13.96 (13.13,14.84) | -0.64 (-0.74,-0.54) |
| Egypt | 13.22 (12.45,13.98) | 10.66 (10.04,11.27) | -0.88 (-1.1,-0.66) |
| El Salvador | 20.76 (19.52,22.06) | 13.57 (12.76,14.39) | -1.38 (-1.46,-1.3) |
| Equatorial Guinea | 34.26 (32.38,36.28) | 17.9 (16.88,18.98) | -2.48 (-2.86,-2.1) |
| Eritrea | 43.79 (41.26,46.55) | 31.37 (29.61,33.46) | -0.89 (-0.98,-0.79) |
| Estonia | 9.88 (9.33,10.43) | 9 (8.55,9.48) | -0.34 (-0.42,-0.27) |
| Eswatini | 20.06 (18.85,21.22) | 16.31 (15.4,17.24) | -0.62 (-0.73,-0.52) |
| Ethiopia | 52.24 (49.11,55.83) | 34 (31.94,36.12) | -1.36 (-1.63,-1.09) |
| Fiji | 7.93 (7.47,8.37) | 8 (7.57,8.45) | 0.38 (0.25,0.51) |
| Finland | 9.01 (8.52,9.52) | 7.59 (7.21,8) | -0.61 (-0.67,-0.55) |
| France | 5.75 (5.42,6.1) | 4.97 (4.71,5.26) | -0.34 (-0.42,-0.27) |
| Gabon | 19.12 (17.85,20.19) | 17.72 (16.7,18.78) | -0.09 (-0.15,-0.02) |
| Gambia | 30.04 (28.34,31.85) | 22.22 (20.95,23.59) | -0.96 (-1.05,-0.87) |
| Georgia | 15.25 (14.4,16.1) | 15.3 (14.57,16.13) | 0.12 (0.03,0.2) |
| Germany | 8.01 (7.57,8.44) | 6.25 (5.86,6.64) | -0.82 (-0.9,-0.75) |
| Ghana | 23.99 (22.56,25.37) | 18.47 (17.46,19.51) | -0.79 (-0.84,-0.74) |
| Greece | 8.64 (8.13,9.17) | 9.15 (8.61,9.72) | 0.48 (0.25,0.71) |
| Greenland | 9.65 (9.14,10.14) | 8.01 (7.58,8.49) | -0.62 (-0.75,-0.49) |
| Grenada | 19.46 (18.43,20.49) | 17.62 (16.7,18.6) | -0.16 (-0.21,-0.11) |
| Guam | 5.71 (5.41,6.03) | 7.15 (6.76,7.54) | 1.02 (0.84,1.2) |
| Guatemala | 28.93 (27.28,30.7) | 19.18 (18.06,20.32) | -1.12 (-1.27,-0.98) |
| Guinea | 32.24 (30.25,34.04) | 24.61 (23.25,26.26) | -0.74 (-0.86,-0.62) |
| Guinea-Bissau | 34.24 (32.37,36.22) | 26.32 (24.81,27.89) | -0.76 (-0.86,-0.67) |
| Guyana | 24.86 (23.48,26.26) | 20.3 (19.23,21.41) | -0.53 (-0.62,-0.44) |
| Haiti | 43.47 (41.21,45.62) | 32.39 (30.63,34.16) | -0.91 (-0.99,-0.83) |
| Honduras | 24.91 (23.43,26.34) | 15.92 (15.01,16.92) | -1.38 (-1.44,-1.33) |
| Hungary | 16.79 (15.86,17.77) | 14.01 (13.26,14.77) | -0.53 (-0.59,-0.47) |
| Iceland | 6.1 (5.75,6.46) | 6.28 (5.93,6.66) | 0.42 (0.27,0.56) |
| India | 18.37 (17.36,19.42) | 16.11 (15.24,17.05) | -0.51 (-0.61,-0.41) |
| Indonesia | 22.87 (21.55,24.28) | 13.81 (13,14.59) | -1.54 (-1.64,-1.44) |
| Iran (Islamic Republic of) | 15.36 (14.31,16.31) | 12.66 (11.91,13.46) | -0.63 (-0.71,-0.56) |
| Iraq | 11.43 (10.77,12.07) | 8.78 (8.28,9.34) | -0.97 (-1.14,-0.79) |
| Ireland | 7.08 (6.68,7.52) | 5.81 (5.48,6.15) | -0.67 (-0.73,-0.61) |
| Israel | 8.68 (8.21,9.12) | 7.26 (6.84,7.69) | -0.56 (-0.63,-0.49) |
| Italy | 13.65 (13.41,13.87) | 10.19 (10.01,10.35) | -0.93 (-0.95,-0.9) |
| Jamaica | 20.41 (19.37,21.62) | 16.99 (16.18,17.98) | -0.39 (-0.47,-0.31) |
| Japan | 10.13 (10.02,10.25) | 9.57 (9.45,9.68) | -0.1 (-0.15,-0.05) |
| Jordan | 6.31 (5.92,6.73) | 5.02 (4.7,5.38) | -0.9 (-0.97,-0.84) |
| Kazakhstan | 16.21 (15.3,17.08) | 14.72 (13.92,15.55) | -0.35 (-0.38,-0.32) |
| Kenya | 39.53 (36.97,42.19) | 27.98 (26.44,29.66) | -0.93 (-1.08,-0.78) |
| Kiribati | 13.46 (12.72,14.21) | 12.52 (11.85,13.25) | 0.1 (-0.01,0.21) |
| Kuwait | 10.71 (10.11,11.35) | 14.71 (13.92,15.53) | 1.25 (1.07,1.43) |
| Kyrgyzstan | 17.33 (16.42,18.26) | 16.17 (15.19,17.01) | -0.13 (-0.25,-0.02) |
| Lao People's Democratic Republic | 36.12 (34.07,38.33) | 19.33 (18.2,20.53) | -2.04 (-2.23,-1.85) |
| Latvia | 11.5 (10.85,12.18) | 10.17 (9.67,10.75) | -0.33 (-0.4,-0.26) |
| Lebanon | 7.16 (6.74,7.65) | 5.7 (5.39,6.04) | -0.9 (-1.01,-0.79) |
| Lesotho | 22.74 (21.44,24.04) | 18.25 (17.23,19.23) | -0.61 (-0.7,-0.52) |
| Liberia | 33.85 (31.85,35.89) | 24.41 (22.87,25.91) | -1.26 (-1.41,-1.11) |
| Libya | 7.75 (7.31,8.19) | 6.74 (6.35,7.14) | -0.63 (-0.75,-0.52) |
| Lithuania | 11.13 (10.54,11.77) | 9.66 (9.15,10.23) | -0.34 (-0.43,-0.24) |
| Luxembourg | 5.27 (4.98,5.57) | 4.68 (4.41,4.94) | -0.26 (-0.36,-0.16) |
| Madagascar | 43.85 (41.27,46.32) | 34.96 (32.98,37.11) | -0.64 (-0.79,-0.5) |
| Malawi | 45.77 (42.86,48.7) | 32.52 (30.58,34.4) | -1.21 (-1.37,-1.05) |
| Malaysia | 15.73 (14.88,16.61) | 11.26 (10.64,11.9) | -0.97 (-1.05,-0.89) |
| Maldives | 21.21 (20.03,22.37) | 10.94 (10.3,11.6) | -2.28 (-2.44,-2.13) |
| Mali | 35.99 (33.73,38.24) | 27.27 (25.53,28.99) | -0.85 (-0.91,-0.79) |
| Malta | 8.69 (8.18,9.21) | 6.55 (6.17,6.91) | -0.89 (-0.98,-0.81) |
| Marshall Islands | 9.39 (8.88,9.94) | 9.73 (9.2,10.28) | 0.45 (0.34,0.55) |
| Mauritania | 25.32 (23.97,26.89) | 21.13 (19.93,22.42) | -0.47 (-0.51,-0.44) |
| Mauritius | 13.27 (12.53,14.11) | 11.17 (10.59,11.79) | -0.5 (-0.56,-0.44) |
| Mexico | 34.63 (34.19,35.06) | 22.93 (22.64,23.21) | -1.42 (-1.49,-1.36) |
| Micronesia (Federated States of) | 10.89 (10.32,11.51) | 9.64 (9.1,10.24) | -0.11 (-0.2,-0.03) |
| Monaco | 6.06 (5.74,6.4) | 4.89 (4.62,5.17) | -0.69 (-0.79,-0.58) |
| Mongolia | 21.06 (19.86,22.21) | 19.85 (18.86,21) | -0.23 (-0.32,-0.13) |
| Montenegro | 15.97 (15.13,16.88) | 13.86 (13.12,14.62) | -0.63 (-0.7,-0.57) |
| Morocco | 9.02 (8.52,9.55) | 7.15 (6.77,7.61) | -0.85 (-0.93,-0.77) |
| Mozambique | 53.02 (49.98,56.47) | 35.15 (33.09,37.51) | -1.23 (-1.35,-1.11) |
| Myanmar | 32.86 (31.08,34.98) | 18.03 (16.94,19.14) | -1.98 (-2.13,-1.83) |
| Namibia | 19.71 (18.63,21.02) | 15.89 (15,16.87) | -0.65 (-0.74,-0.55) |
| Nauru | 7.1 (6.72,7.5) | 7.98 (7.53,8.47) | 0.57 (0.42,0.72) |
| Nepal | 15.78 (14.92,16.73) | 10.11 (9.55,10.74) | -1.41 (-1.46,-1.35) |
| Netherlands | 6.99 (6.6,7.38) | 5.78 (5.45,6.13) | -0.6 (-0.65,-0.56) |
| New Zealand | 14.58 (13.99,15.25) | 11.67 (11.18,12.2) | -0.69 (-0.75,-0.62) |
| Nicaragua | 21.95 (20.69,23.32) | 13.96 (13.18,14.84) | -1.42 (-1.45,-1.4) |
| Niger | 38.33 (35.95,40.77) | 35.21 (33.11,37.66) | -0.26 (-0.35,-0.16) |
| Nigeria | 27.08 (25.5,28.71) | 22.87 (21.5,24.29) | -0.57 (-0.65,-0.49) |
| Niue | 6.74 (6.39,7.11) | 7.31 (6.9,7.74) | 0.39 (0.34,0.44) |
| North Macedonia | 21.79 (20.56,22.99) | 19.35 (18.39,20.37) | -0.49 (-0.56,-0.41) |
| Northern Mariana Islands | 6.01 (5.7,6.37) | 7.67 (7.29,8.1) | 1.06 (0.96,1.17) |
| Norway | 5.77 (5.66,5.88) | 5.04 (4.94,5.15) | -0.46 (-0.5,-0.42) |
| Oman | 8.26 (7.75,8.77) | 6.89 (6.5,7.31) | -0.71 (-0.84,-0.57) |
| Pakistan | 15.69 (14.84,16.53) | 12.72 (12,13.44) | -0.59 (-0.63,-0.55) |
| Palau | 6.4 (6.05,6.76) | 7.79 (7.34,8.28) | 0.93 (0.83,1.04) |
| Palestine | 8.39 (7.85,8.93) | 6.37 (5.98,6.75) | -0.95 (-1.1,-0.8) |
| Panama | 13.29 (12.54,14.08) | 10.31 (9.72,10.94) | -0.63 (-0.71,-0.56) |
| Papua New Guinea | 12.96 (12.28,13.68) | 10.69 (10.08,11.33) | -0.44 (-0.53,-0.36) |
| Paraguay | 17.06 (16.14,18.07) | 11.92 (11.27,12.61) | -1.11 (-1.17,-1.06) |
| Peru | 22.05 (20.9,23.31) | 14.84 (13.91,15.77) | -1.22 (-1.27,-1.17) |
| Philippines | 16.5 (15.54,17.45) | 10.51 (9.9,11.17) | -1.38 (-1.55,-1.22) |
| Poland | 5.63 (5.54,5.74) | 4.37 (4.29,4.45) | -0.91 (-1,-0.83) |
| Portugal | 5.28 (5.01,5.57) | 3.22 (3.04,3.4) | -1.76 (-1.87,-1.65) |
| Puerto Rico | 13.91 (13.19,14.74) | 11.72 (11.1,12.31) | -0.48 (-0.55,-0.42) |
| Qatar | 9.3 (8.75,9.85) | 5.85 (5.49,6.22) | -1.54 (-1.72,-1.36) |
| Republic of Korea | 10.85 (10.25,11.46) | 8.92 (8.43,9.44) | -0.63 (-0.77,-0.5) |
| Republic of Moldova | 13.6 (12.91,14.37) | 11.27 (10.64,11.87) | -0.6 (-0.69,-0.51) |
| Romania | 16.33 (15.51,17.31) | 14.2 (13.47,14.97) | -0.45 (-0.48,-0.43) |
| Russian Federation | 11.38 (10.79,12) | 10.11 (9.57,10.66) | -0.37 (-0.45,-0.3) |
| Rwanda | 42.2 (39.68,44.71) | 28.33 (26.65,29.96) | -1.51 (-1.71,-1.3) |
| Saint Kitts and Nevis | 15.54 (14.76,16.44) | 13.36 (12.66,14.04) | -0.4 (-0.44,-0.37) |
| Saint Lucia | 19.36 (18.4,20.47) | 17.15 (16.33,18.06) | -0.27 (-0.31,-0.23) |
| Saint Vincent and the Grenadines | 19.46 (18.38,20.56) | 17.67 (16.72,18.7) | -0.25 (-0.28,-0.22) |
| Samoa | 9.67 (9.14,10.23) | 10.08 (9.53,10.65) | 0.58 (0.43,0.72) |
| San Marino | 5.3 (5.02,5.63) | 5.72 (5.4,6.08) | 0.36 (0.28,0.43) |
| Sao Tome and Principe | 27 (25.46,28.6) | 16.98 (16.01,17.99) | -1.68 (-1.78,-1.57) |
| Saudi Arabia | 10.51 (9.91,11.07) | 10.36 (9.81,11) | -0.12 (-0.3,0.06) |
| Senegal | 27.51 (25.77,29.2) | 21.84 (20.56,23.12) | -0.53 (-0.59,-0.46) |
| Serbia | 18.13 (17.19,19.16) | 13.29 (12.62,14.06) | -1.21 (-1.28,-1.14) |
| Seychelles | 13.33 (12.59,14.08) | 11.51 (10.9,12.14) | -0.48 (-0.53,-0.43) |
| Sierra Leone | 31.19 (29.44,33.15) | 23.74 (22.28,25.32) | -0.91 (-1.12,-0.7) |
| Singapore | 10.12 (9.55,10.74) | 7.38 (6.98,7.76) | -0.91 (-1,-0.82) |
| Slovakia | 18.5 (17.49,19.65) | 16.64 (15.75,17.55) | -0.35 (-0.44,-0.26) |
| Slovenia | 12.66 (11.97,13.38) | 10.1 (9.51,10.64) | -0.85 (-0.92,-0.78) |
| Solomon Islands | 16.99 (16.08,18.01) | 15.62 (14.74,16.56) | 0.11 (-0.03,0.25) |
| Somalia | 56.42 (53,60.14) | 56.13 (52.71,59.44) | 0 (-0.07,0.08) |
| South Africa | 23.09 (22.59,23.59) | 23.4 (22.89,23.94) | -0.03 (-0.11,0.06) |
| South Sudan | 40.68 (38.32,42.91) | 36.94 (34.93,39.18) | -0.27 (-0.34,-0.19) |
| Spain | 5.66 (5.34,6) | 5.39 (5.06,5.69) | 0.06 (-0.06,0.19) |
| Sri Lanka | 18.41 (17.42,19.58) | 12.86 (12.06,13.62) | -1.12 (-1.27,-0.97) |
| Sudan | 16.43 (15.47,17.52) | 9.06 (8.52,9.62) | -2.12 (-2.27,-1.98) |
| Suriname | 23.43 (22.08,24.79) | 18.98 (17.9,19.97) | -0.68 (-0.77,-0.58) |
| Sweden | 6.81 (6.38,7.22) | 7.43 (7.03,7.86) | 0.59 (0.47,0.7) |
| Switzerland | 8.81 (8.31,9.29) | 6.59 (6.24,6.94) | -1.1 (-1.16,-1.05) |
| Syrian Arab Republic | 8.52 (8.04,9.02) | 6.01 (5.64,6.4) | -1.04 (-1.25,-0.83) |
| Taiwan (Province of China) | 10.09 (9.49,10.66) | 9.52 (9.02,10.06) | 0 (-0.08,0.07) |
| Tajikistan | 21.6 (20.48,22.7) | 20.62 (19.51,21.76) | -0.19 (-0.3,-0.08) |
| Thailand | 38.21 (36.04,40.33) | 25.86 (24.44,27.37) | -1.08 (-1.2,-0.96) |
| Timor-Leste | 41.47 (39.23,43.99) | 20.18 (19.02,21.34) | -2.75 (-2.94,-2.55) |
| Togo | 27.6 (26,29.36) | 21.54 (20.2,22.89) | -0.69 (-0.82,-0.56) |
| Tokelau | 6.97 (6.6,7.35) | 7.62 (7.2,8.05) | 0.28 (0.2,0.35) |
| Tonga | 9.24 (8.74,9.79) | 9 (8.51,9.5) | 0.27 (0.16,0.38) |
| Trinidad and Tobago | 19.52 (18.5,20.66) | 18.11 (17.12,19.18) | -0.29 (-0.32,-0.25) |
| Tunisia | 7.67 (7.21,8.1) | 5.74 (5.41,6.09) | -0.99 (-1.08,-0.9) |
| Turkey | 8.15 (7.71,8.62) | 5.32 (5,5.65) | -1.44 (-1.6,-1.29) |
| Turkmenistan | 16.38 (15.45,17.34) | 15.8 (14.94,16.71) | -0.21 (-0.35,-0.08) |
| Tuvalu | 10.75 (10.22,11.31) | 8.77 (8.28,9.25) | -0.39 (-0.51,-0.27) |
| Uganda | 61.5 (57.94,65.3) | 38.34 (36.23,40.51) | -1.64 (-1.76,-1.51) |
| Ukraine | 11.89 (11.21,12.6) | 11.3 (10.68,11.97) | -0.13 (-0.18,-0.07) |
| United Arab Emirates | 8.18 (7.7,8.66) | 6.43 (6.09,6.77) | -0.84 (-0.94,-0.74) |
| United Kingdom | 4.93 (4.89,4.96) | 5.53 (5.49,5.57) | 0.53 (0.41,0.66) |
| United Republic of Tanzania | 40.42 (38.09,42.8) | 28.97 (27.23,30.93) | -1.01 (-1.16,-0.86) |
| United States of America | 7.56 (7.15,8) | 6.54 (6.17,6.89) | -0.5 (-0.52,-0.48) |
| United States Virgin Islands | 15.4 (14.65,16.28) | 14.43 (13.69,15.28) | -0.04 (-0.1,0.01) |
| Uruguay | 12.05 (11.37,12.75) | 11.03 (10.47,11.7) | -0.28 (-0.32,-0.25) |
| Uzbekistan | 17.66 (16.72,18.65) | 18.02 (17.09,19.07) | -0.03 (-0.11,0.05) |
| Vanuatu | 13.83 (12.96,14.66) | 13.4 (12.61,14.2) | 0.22 (0.13,0.31) |
| Venezuela (Bolivarian Republic of) | 13.56 (12.82,14.38) | 11.78 (11.09,12.48) | -0.31 (-0.47,-0.16) |
| Viet Nam | 24.88 (23.45,26.48) | 12.91 (12.2,13.62) | -2.13 (-2.19,-2.08) |
| Yemen | 15.57 (14.65,16.54) | 10.28 (9.66,10.86) | -1.44 (-1.64,-1.24) |
| Zambia | 36.4 (34.4,38.39) | 24.77 (23.36,26.25) | -1.31 (-1.52,-1.09) |
| Zimbabwe | 20.93 (19.55,22.3) | 19.4 (18.31,20.62) | 0.12 (-0.05,0.3) |
