# Supplementary material for: Analysis of global, regional, and national burdens of neonatal encephalopathy from 1990 to 2021: insights from the Global Burden of Disease Study 2021
Source: Front Public Health. 2025 Oct 8;13:1627448. doi: 10.3389/fpubh.2025.1627448 (PMC12540312; doi:10.3389/fpubh.2025.1627448)
Supplement: Supplementary file 3 [file Table_3.doc]

| Location | Incidence | | |
| --- | --- | --- | --- |
|  | Number (95% UI) | | Percentage  change  (95% UI) |
|  | 1990 | 2021 |  |
| Afghanistan | 3503.49 (3285.31, 3710.92) | 5251.72 (4936.82, 5558.37) | 49.9 (41.42, 58.38) |
| Albania | 769.18 (723.27, 809.45) | 186.35 (175.34, 198.27) | -75.77 (-84.09, -67.45) |
| Algeria | 2695.35 (2523.45, 2859.8) | 2626.75 (2472.9, 2787.34) | -2.55 (-11.19, 6.1) |
| American Samoa | 5.7 (5.39, 6.03) | 2.88 (2.74, 3.05) | -49.47 (-57.21, -41.73) |
| Andorra | 1.88 (1.78, 1.99) | 1.51 (1.42, 1.59) | -19.68 (-27.04, -12.32) |
| Angola | 7291.4 (6850.51, 7718.04) | 9724.94 (9179.61, 10329.51) | 33.38 (24.99, 41.76) |
| Antigua and Barbuda | 9.69 (9.17, 10.25) | 7.91 (7.49, 8.34) | -18.37 (-26.23, -10.51) |
| Argentina | 5072.06 (4784.11, 5374.4) | 3587.94 (3403.8, 3806.95) | -29.26 (-37.35, -21.17) |
| Armenia | 647.73 (615.56, 683.4) | 295.04 (279.28, 311.73) | -54.45 (-62.05, -46.85) |
| Australia | 730.31 (688.17, 774.49) | 560.12 (527.85, 595.5) | -23.3 (-31.75, -14.85) |
| Austria | 419.3 (396.39, 443.72) | 329.34 (311.08, 348.92) | -21.45 (-29.51, -13.4) |
| Azerbaijan | 1572.57 (1489.35, 1660.57) | 1049.9 (996.07, 1109.73) | -33.24 (-40.91, -25.56) |
| Bahamas | 39.9 (37.83, 42.31) | 27.62 (26.23, 29.07) | -30.78 (-38.36, -23.2) |
| Bahrain | 79.41 (74.84, 84.19) | 73.9 (69.62, 78.29) | -6.94 (-15.25, 1.38) |
| Bangladesh | 40081.23 (37950.22, 42797.14) | 16835.81 (15922.97, 17833) | -58 (-66.29, -49.71) |
| Barbados | 32.95 (31.27, 34.89) | 20 (18.93, 21.12) | -39.3 (-47.05, -31.55) |
| Belarus | 842.65 (797.31, 886.62) | 426.42 (404.75, 451.8) | -49.4 (-57.04, -41.75) |
| Belgium | 275.86 (260.38, 291.45) | 185.8 (175.85, 195.96) | -32.65 (-40.46, -24.83) |
| Belize | 84.5 (79.95, 89.53) | 85.63 (81.1, 90.8) | 1.34 (-6.66, 9.34) |
| Benin | 3437.93 (3250.88, 3662.3) | 5581.12 (5272.76, 5926.3) | 62.34 (53.97, 70.71) |
| Bermuda | 5.53 (5.24, 5.84) | 2.6 (2.45, 2.76) | -52.98 (-61.02, -44.94) |
| Bhutan | 186.91 (177.25, 197.1) | 62.05 (58.61, 65.73) | -66.8 (-74.63, -58.98) |
| Bolivia (Plurinational State of) | 2781.71 (2619.32, 2954.27) | 1945.44 (1837.48, 2065.07) | -30.06 (-38.46, -21.67) |
| Bosnia and Herzegovina | 720.16 (678.67, 759) | 230.68 (218.27, 244.16) | -67.97 (-75.88, -60.06) |
| Botswana | 406.93 (384.12, 433.01) | 346.76 (326.92, 370.49) | -14.79 (-23.48, -6.1) |
| Brazil | 16314.4 (16011.06, 16621.04) | 12920.3 (12727.75, 13128.44) | -20.8 (-23.23, -18.38) |
| Brunei Darussalam | 27.41 (26, 29.2) | 22.23 (21.08, 23.4) | -18.9 (-26.74, -11.06) |
| Bulgaria | 845.63 (800.32, 894.9) | 446.17 (422.39, 470.64) | -47.24 (-55.02, -39.46) |
| Burkina Faso | 7646.05 (7195.08, 8109.94) | 12378.7 (11672.77, 13157.91) | 61.9 (53.42, 70.37) |
| Burundi | 5610.9 (5308.01, 5983.24) | 8869.84 (8343.14, 9379.05) | 58.08 (49.7, 66.47) |
| Cabo Verde | 141.03 (132.67, 150.4) | 79.08 (74.57, 83.67) | -43.93 (-52.44, -35.41) |
| Cambodia | 7935.89 (7479.16, 8422.38) | 3314.06 (3140.27, 3520.33) | -58.24 (-66.5, -49.98) |
| Cameroon | 5215.75 (4918.3, 5531.39) | 10359.66 (9749.95, 10978.4) | 98.62 (90.27, 106.97) |
| Canada | 1281.77 (1209.22, 1357.42) | 1021.84 (967.4, 1074.57) | -20.28 (-28.08, -12.47) |
| Central African Republic | 2026.62 (1910.72, 2163.61) | 2730.05 (2577.79, 2906.86) | 34.71 (26.03, 43.38) |
| Chad | 5375.24 (5032.49, 5727.8) | 11791.97 (11130.07, 12530.79) | 119.38 (110.59, 128.16) |
| Chile | 2876.41 (2718.05, 3039.6) | 1539.61 (1449.08, 1622.8) | -46.47 (-54.42, -38.53) |
| China | 197585.09 (185783.26, 208753.85) | 74198.78 (70060.55, 78261.43) | -62.45 (-70.47, -54.43) |
| Colombia | 6777.76 (6425.16, 7160.13) | 3928.55 (3699.06, 4179.08) | -42.04 (-50.21, -33.87) |
| Comoros | 387.64 (363.29, 414.07) | 222.17 (209.51, 237.34) | -42.69 (-51.75, -33.63) |
| Congo | 1042.17 (982.58, 1103.11) | 1129.74 (1065.51, 1205.15) | 8.4 (-0.06, 16.87) |
| Cook Islands | 1.24 (1.17, 1.31) | 0.65 (0.61, 0.68) | -47.58 (-56.32, -38.84) |
| Costa Rica | 492.2 (464.27, 521.14) | 290.37 (273.58, 307.09) | -41.01 (-49.17, -32.84) |
| Croatia | 456.48 (435.4, 480.8) | 251.39 (237.56, 264.55) | -44.93 (-52.25, -37.61) |
| Cuba | 1327.21 (1258.29, 1400.88) | 673.36 (635.29, 710.07) | -49.26 (-56.99, -41.54) |
| Cyprus | 49.77 (47.16, 52.74) | 41.48 (39.19, 44.04) | -16.66 (-24.76, -8.56) |
| Czechia | 1207.07 (1142.7, 1279.69) | 756.72 (716.05, 800.15) | -37.31 (-45.25, -29.37) |
| Côte d'Ivoire | 7284.92 (6872.87, 7779.79) | 9718.72 (9193.97, 10292.97) | 33.41 (25, 41.82) |
| Democratic People's Republic of Korea | 4749.41 (4474.74, 5026.1) | 2387.74 (2249.83, 2527.61) | -49.73 (-57.94, -41.51) |
| Democratic Republic of the Congo | 24379.97 (22913.49, 25918.9) | 31193.17 (29391.52, 33247.07) | 27.95 (19.22, 36.67) |
| Denmark | 259.73 (245.68, 274.34) | 219.36 (208.05, 232.32) | -15.54 (-23.35, -7.73) |
| Djibouti | 185.91 (174.88, 196.76) | 326.46 (309.25, 346.33) | 75.6 (67.42, 83.78) |
| Dominica | 17.14 (16.29, 18.13) | 5.07 (4.79, 5.35) | -70.42 (-78.05, -62.79) |
| Dominican Republic | 2343.33 (2222.01, 2478.55) | 1749.98 (1659.3, 1844.32) | -25.32 (-32.93, -17.71) |
| Ecuador | 2607.55 (2467.83, 2758.43) | 2150.19 (2022.42, 2285.58) | -17.54 (-25.82, -9.26) |
| Egypt | 11939.31 (11243.73, 12627.57) | 13184.56 (12420.11, 13945.68) | 10.43 (2.24, 18.62) |
| El Salvador | 1734.55 (1631.35, 1843.3) | 758.68 (713.38, 804.52) | -56.26 (-64.83, -47.69) |
| Equatorial Guinea | 337.38 (318.94, 357.34) | 321.71 (303.46, 341.15) | -4.64 (-12.81, 3.52) |
| Eritrea | 3108.88 (2929.27, 3305.1) | 2936.42 (2771.86, 3132.29) | -5.55 (-14.16, 3.07) |
| Estonia | 102.15 (96.45, 107.86) | 56.17 (53.35, 59.2) | -45.01 (-52.64, -37.38) |
| Eswatini | 310.83 (292.05, 328.74) | 226.22 (213.59, 239.15) | -27.22 (-35.39, -19.05) |
| Ethiopia | 61018.73 (57361.2, 65204.46) | 56768.73 (53326.37, 60314.33) | -6.97 (-15.86, 1.93) |
| Fiji | 71.7 (67.58, 75.72) | 69.4 (65.65, 73.33) | -3.21 (-11.14, 4.73) |
| Finland | 284.41 (269.15, 300.44) | 176.37 (167.56, 186.04) | -37.99 (-45.58, -30.4) |
| France | 2120.12 (1995.9, 2246.37) | 1648.89 (1562.2, 1745.55) | -22.23 (-30.34, -14.11) |
| Gabon | 326.56 (304.82, 344.82) | 365.93 (344.82, 387.73) | 12.06 (3.58, 20.53) |
| Gambia | 651 (614.18, 690.17) | 823.5 (776.29, 874.18) | 26.5 (18.17, 34.83) |
| Georgia | 632.55 (597.34, 667.74) | 328.69 (312.92, 346.46) | -48.04 (-55.59, -40.49) |
| Germany | 3390.92 (3206.07, 3575.36) | 2362.27 (2215.9, 2509.49) | -30.34 (-38.6, -22.07) |
| Ghana | 6944.83 (6531.06, 7344.26) | 8578.52 (8109.56, 9063.77) | 23.52 (15.45, 31.6) |
| Greece | 434.8 (409.47, 461.84) | 363.67 (342.39, 386.65) | -16.36 (-24.92, -7.8) |
| Greenland | 5.58 (5.29, 5.87) | 2.99 (2.83, 3.17) | -46.42 (-54.33, -38.51) |
| Grenada | 22.55 (21.35, 23.74) | 11.42 (10.82, 12.06) | -49.36 (-56.99, -41.72) |
| Guam | 10.38 (9.84, 10.96) | 9.07 (8.57, 9.56) | -12.62 (-20.31, -4.93) |
| Guatemala | 4805.69 (4531.88, 5099.5) | 2732.52 (2574.07, 2895.95) | -43.14 (-51.48, -34.8) |
| Guinea | 4496.57 (4218.74, 4747.67) | 5865.14 (5541, 6258.43) | 30.44 (21.95, 38.92) |
| Guinea-Bissau | 754.36 (713.15, 797.89) | 905.15 (853.21, 959.13) | 19.99 (11.88, 28.1) |
| Guyana | 311.11 (293.9, 328.66) | 145.56 (137.85, 153.48) | -53.21 (-60.96, -45.46) |
| Haiti | 5265.75 (4991.23, 5526.21) | 5311.02 (5023.02, 5601.43) | 0.86 (-6.59, 8.31) |
| Honduras | 2139.67 (2012.58, 2262.09) | 1699.7 (1602.49, 1806.84) | -20.56 (-28.94, -12.19) |
| Hungary | 1014.83 (958.42, 1073.57) | 597.25 (565.57, 629.77) | -41.15 (-48.96, -33.33) |
| Iceland | 13.59 (12.81, 14.37) | 13.95 (13.17, 14.8) | 2.65 (-5.6, 10.9) |
| India | 221146.04 (209058.65, 233806.81) | 169064.43 (159957.63, 178943.38) | -23.55 (-31.48, -15.62) |
| Indonesia | 51501.35 (48531.68, 54677.02) | 29254.88 (27546.75, 30905.23) | -43.2 (-51.48, -34.92) |
| Iran (Islamic Republic of) | 11419.47 (10641.41, 12128.96) | 6272.77 (5901.14, 6665.98) | -45.07 (-53.99, -36.15) |
| Iraq | 3794.09 (3574.04, 4005.32) | 3493.19 (3293.53, 3716.26) | -7.93 (-16.23, 0.37) |
| Ireland | 185.51 (175.07, 196.95) | 157.64 (148.64, 166.88) | -15.02 (-23.28, -6.77) |
| Israel | 438.81 (415.1, 461.51) | 644.09 (607.1, 682.55) | 46.78 (38.89, 54.67) |
| Italy | 3672.4 (3608.87, 3731.73) | 1976.94 (1943.12, 2008.01) | -46.17 (-48.51, -43.82) |
| Jamaica | 564.79 (536.07, 598.32) | 269.44 (256.5, 285.04) | -52.29 (-59.94, -44.65) |
| Japan | 6068.35 (6000.63, 6140.59) | 3956.08 (3905.82, 4003.05) | -34.81 (-36.49, -33.12) |
| Jordan | 403.47 (378.53, 430.29) | 519.57 (486.96, 557.09) | 28.78 (19.47, 38.08) |
| Kazakhstan | 2878.03 (2716.38, 3032.03) | 2888.31 (2731.78, 3051.1) | 0.36 (-7.43, 8.14) |
| Kenya | 18656.84 (17450.67, 19914.25) | 15853.32 (14978.75, 16799.85) | -15.03 (-23.78, -6.28) |
| Kiribati | 17.47 (16.51, 18.44) | 17.6 (16.66, 18.62) | 0.74 (-7.08, 8.57) |
| Kuwait | 172.62 (162.94, 182.86) | 352.61 (333.51, 372.21) | 104.27 (96.31, 112.23) |
| Kyrgyzstan | 1100.68 (1043.47, 1159.9) | 1214.74 (1141.45, 1278.2) | 10.36 (2.64, 18.09) |
| Lao People's Democratic Republic | 3011.53 (2840.36, 3195.66) | 1628.15 (1532.9, 1729.37) | -45.94 (-54.37, -37.5) |
| Latvia | 203.43 (191.92, 215.31) | 82.46 (78.36, 87.13) | -59.47 (-67.3, -51.63) |
| Lebanon | 292.72 (275.85, 313.03) | 216.33 (204.62, 229.2) | -26.1 (-34.62, -17.58) |
| Lesotho | 583.52 (550.2, 617.05) | 370.05 (349.33, 389.89) | -36.58 (-44.51, -28.66) |
| Liberia | 1828.02 (1720.05, 1938.32) | 1904.12 (1784.67, 2021.83) | 4.16 (-4.46, 12.79) |
| Libya | 484.31 (457.01, 512.2) | 259.62 (244.66, 274.95) | -46.39 (-54.55, -38.24) |
| Lithuania | 301.72 (285.64, 319.17) | 110.58 (104.74, 117.03) | -63.35 (-71.21, -55.49) |
| Luxembourg | 12.77 (12.07, 13.48) | 15.21 (14.36, 16.08) | 19.11 (11.19, 27.02) |
| Madagascar | 10970.02 (10323.91, 11588.83) | 14161.28 (13357.51, 15032.34) | 29.09 (20.83, 37.35) |
| Malawi | 10976.4 (10278.11, 11678.15) | 8873.96 (8342.66, 9386.35) | -19.15 (-27.83, -10.48) |
| Malaysia | 3757.72 (3556.26, 3967.85) | 2584.15 (2442.11, 2730.49) | -31.23 (-39.05, -23.41) |
| Maldives | 90.35 (85.32, 95.29) | 31.63 (29.77, 33.54) | -64.99 (-73.1, -56.88) |
| Mali | 7711.6 (7227.62, 8193.44) | 13917.1 (13030.32, 14793.67) | 80.47 (71.56, 89.38) |
| Malta | 23.24 (21.87, 24.64) | 13.7 (12.92, 14.46) | -41.05 (-49.23, -32.87) |
| Marshall Islands | 6.75 (6.38, 7.14) | 5.33 (5.04, 5.63) | -21.04 (-28.84, -13.24) |
| Mauritania | 1050.95 (995.11, 1116.35) | 1370.63 (1292.96, 1454.72) | 30.42 (22.17, 38.67) |
| Mauritius | 147.4 (139.2, 156.72) | 68.13 (64.56, 71.91) | -53.78 (-61.8, -45.76) |
| Mexico | 42180.71 (41652.82, 42707.83) | 20777.95 (20514.82, 21036.58) | -50.74 (-52.51, -48.97) |
| Micronesia (Federated States of) | 16.47 (15.61, 17.42) | 8.62 (8.14, 9.16) | -47.66 (-55.72, -39.61) |
| Monaco | 0.78 (0.74, 0.83) | 0.75 (0.71, 0.79) | -3.85 (-11.1, 3.4) |
| Mongolia | 729.88 (688.26, 769.56) | 727.83 (691.48, 770.04) | -0.28 (-8.04, 7.47) |
| Montenegro | 76.4 (72.36, 80.78) | 46.86 (44.33, 49.43) | -38.66 (-46.41, -30.92) |
| Morocco | 3417.84 (3228.43, 3617.99) | 2189.85 (2072.57, 2329.49) | -35.93 (-44.11, -27.75) |
| Mozambique | 15532.39 (14641.54, 16542.36) | 18630.94 (17538.68, 19884.88) | 19.95 (11.17, 28.73) |
| Myanmar | 17613.84 (16658.22, 18746.62) | 9274.43 (8711.64, 9843.18) | -47.35 (-55.85, -38.84) |
| Namibia | 484.72 (458.24, 517.05) | 437.57 (413.04, 464.4) | -9.73 (-18.17, -1.29) |
| Nauru | 1.2 (1.13, 1.27) | 1.11 (1.05, 1.18) | -7.5 (-15.91, 0.91) |
| Nepal | 5952.86 (5627.64, 6309.57) | 3114.6 (2942.97, 3309.42) | -47.68 (-55.89, -39.47) |
| Netherlands | 664.29 (627.21, 701.68) | 494.32 (466.39, 524.16) | -25.59 (-33.69, -17.49) |
| New Zealand | 422.82 (405.47, 442.03) | 345.28 (330.81, 361.01) | -18.34 (-24.49, -12.19) |
| Nicaragua | 1499.01 (1413.28, 1592.56) | 858.78 (811.05, 912.79) | -42.71 (-51.13, -34.29) |
| Niger | 8197.42 (7687.63, 8718.01) | 19957.92 (18767.78, 21348.92) | 143.47 (134.45, 152.48) |
| Nigeria | 53046.13 (49962.36, 56241.4) | 90497.7 (85073.54, 96088.37) | 70.6 (62.11, 79.09) |
| Niue | 0.15 (0.14, 0.16) | 0.08 (0.08, 0.09) | -46.67 (-59.73, -33.6) |
| North Macedonia | 355.54 (335.42, 374.97) | 175.73 (167.05, 185) | -50.57 (-58.13, -43.02) |
| Northern Mariana Islands | 3.55 (3.36, 3.76) | 2.2 (2.09, 2.32) | -38.03 (-45.71, -30.34) |
| Norway | 165.05 (161.96, 168.32) | 134.94 (132.22, 137.74) | -18.24 (-21.05, -15.43) |
| Oman | 285.02 (267.61, 302.64) | 262.01 (247.17, 277.76) | -8.07 (-16.55, 0.4) |
| Pakistan | 31645.33 (29941.91, 33344.02) | 38007.34 (35837.21, 40163.07) | 20.1 (12.28, 27.93) |
| Palau | 0.96 (0.91, 1.02) | 0.67 (0.63, 0.71) | -30.21 (-38.68, -21.74) |
| Palestine | 355.01 (332.16, 377.65) | 367.34 (345.16, 389.38) | 3.47 (-5.32, 12.26) |
| Panama | 376.94 (355.69, 399.37) | 349.44 (329.48, 370.99) | -7.3 (-15.59, 1) |
| Papua New Guinea | 916.55 (868.47, 967.34) | 1751.17 (1652.37, 1856.87) | 91.06 (83.11, 99.01) |
| Paraguay | 1059.42 (1002.54, 1121.96) | 738.74 (698.27, 781.4) | -30.27 (-38.23, -22.31) |
| Peru | 6810.56 (6457.48, 7199.09) | 4797.86 (4495.84, 5099.89) | -29.55 (-37.88, -21.23) |
| Philippines | 16000.82 (15068.91, 16919.61) | 11469.07 (10801.4, 12183.04) | -28.32 (-36.67, -19.97) |
| Poland | 1479.68 (1456.02, 1507.32) | 717.82 (705.36, 730.94) | -51.49 (-53.98, -49) |
| Portugal | 292.3 (277.4, 308.51) | 127.21 (120.16, 134.38) | -56.48 (-64.2, -48.76) |
| Puerto Rico | 437.93 (415.22, 463.99) | 103.43 (97.96, 108.66) | -76.38 (-83.98, -68.78) |
| Qatar | 50.89 (47.88, 53.93) | 108.58 (101.92, 115.3) | 113.36 (104.81, 121.91) |
| Republic of Korea | 3505.79 (3309.5, 3701.53) | 1145.76 (1083.68, 1212.55) | -67.32 (-75.25, -59.39) |
| Republic of Moldova | 518.05 (492.01, 547.61) | 152.96 (144.44, 161.18) | -70.47 (-78.14, -62.81) |
| Romania | 2371.34 (2251.41, 2513.19) | 1217.62 (1154.62, 1283.56) | -48.65 (-56.3, -41) |
| Russian Federation | 10831.37 (10270.83, 11414.35) | 6582.67 (6227.54, 6941.41) | -39.23 (-46.79, -31.66) |
| Rwanda | 6437.82 (6053.91, 6821.46) | 5016.99 (4718.87, 5305.26) | -22.07 (-30.42, -13.72) |
| Saint Kitts and Nevis | 6.94 (6.59, 7.35) | 3.73 (3.53, 3.92) | -46.25 (-53.76, -38.74) |
| Saint Lucia | 33.15 (31.52, 35.05) | 13.87 (13.21, 14.61) | -58.16 (-65.52, -50.8) |
| Saint Vincent and the Grenadines | 24.05 (22.72, 25.42) | 11.06 (10.47, 11.71) | -54.01 (-62, -46.03) |
| Samoa | 25.03 (23.67, 26.49) | 29.92 (28.3, 31.61) | 19.54 (11.66, 27.41) |
| San Marino | 0.62 (0.59, 0.66) | 0.63 (0.6, 0.67) | 1.61 (-7.26, 10.48) |
| Sao Tome and Principe | 59.88 (56.47, 63.43) | 40.2 (37.91, 42.6) | -32.87 (-41.12, -24.61) |
| Saudi Arabia | 2580.25 (2433.09, 2719.15) | 2306.28 (2183.78, 2450.26) | -10.62 (-18.62, -2.61) |
| Senegal | 4559.94 (4271.45, 4839.49) | 5036.01 (4740.52, 5331.18) | 10.44 (1.89, 19) |
| Serbia | 1199.16 (1136.65, 1267.01) | 429.11 (407.47, 453.98) | -64.22 (-71.89, -56.54) |
| Seychelles | 10.54 (9.95, 11.13) | 8.73 (8.27, 9.21) | -17.17 (-24.93, -9.42) |
| Sierra Leone | 3036.11 (2865.53, 3226.49) | 3436.91 (3225.45, 3665.69) | 13.2 (4.46, 21.94) |
| Singapore | 245.04 (231.41, 260.09) | 195.37 (184.8, 205.35) | -20.27 (-28.14, -12.4) |
| Slovakia | 705.63 (667.21, 749.64) | 445.72 (421.83, 470.06) | -36.83 (-44.79, -28.87) |
| Slovenia | 135.45 (127.98, 143.13) | 90.29 (85.02, 95.13) | -33.34 (-41.25, -25.43) |
| Solomon Islands | 111.22 (105.23, 117.88) | 152.72 (144.07, 161.85) | 37.31 (29.17, 45.46) |
| Somalia | 10468.26 (9832.42, 11157.79) | 25869.54 (24293.31, 27394.01) | 147.12 (138.41, 155.84) |
| South Africa | 11619.14 (11364.32, 11869.21) | 11061.97 (10818.88, 11317.64) | -4.8 (-7.93, -1.66) |
| South Sudan | 4992.97 (4702.97, 5265.77) | 6771.18 (6402.34, 7181.78) | 35.61 (27.56, 43.67) |
| Spain | 1084.46 (1024.52, 1150.63) | 868.77 (815.74, 918.26) | -19.89 (-28.17, -11.61) |
| Sri Lanka | 3176.5 (3005.95, 3377.71) | 1862.8 (1747.96, 1972.85) | -41.36 (-49.76, -32.95) |
| Sudan | 6890.75 (6488.64, 7347.48) | 4994.07 (4694.33, 5303.85) | -27.53 (-36.25, -18.8) |
| Suriname | 102.98 (97.07, 108.95) | 80.96 (76.39, 85.21) | -21.38 (-29.32, -13.45) |
| Sweden | 406.5 (381, 430.68) | 407.65 (385.8, 431.33) | 0.28 (-7.99, 8.56) |
| Switzerland | 356.59 (336.12, 376.07) | 278.76 (263.95, 293.62) | -21.83 (-29.55, -14.1) |
| Syrian Arab Republic | 1878.49 (1772.68, 1989.66) | 567.31 (532.46, 603.83) | -69.8 (-78.34, -61.26) |
| Taiwan (Province of China) | 1577.52 (1484.8, 1667.77) | 720.81 (682.8, 761.77) | -54.31 (-62.29, -46.33) |
| Tajikistan | 2160.79 (2049.07, 2271.26) | 2803.12 (2653.2, 2958.72) | 29.73 (22.23, 37.22) |
| Thailand | 19050.48 (17966.14, 20105.55) | 6668.36 (6303.21, 7059.04) | -65 (-72.97, -57.02) |
| Timor-Leste | 691.67 (654.31, 733.74) | 395.25 (372.48, 417.91) | -42.86 (-50.98, -34.73) |
| Togo | 2123.36 (2000.41, 2258.79) | 2506.44 (2350.3, 2663.41) | 18.04 (9.32, 26.76) |
| Tokelau | 0.12 (0.12, 0.13) | 0.06 (0.06, 0.07) | -50 (-50, -50) |
| Tonga | 14.42 (13.65, 15.28) | 12.84 (12.15, 13.55) | -10.96 (-18.88, -3.03) |
| Trinidad and Tobago | 224 (212.3, 237.08) | 129.22 (122.15, 136.88) | -42.31 (-50.26, -34.37) |
| Tunisia | 808.62 (760.48, 854.06) | 460.3 (433.44, 488.12) | -43.08 (-51.37, -34.78) |
| Turkey | 5795.32 (5480.37, 6125.02) | 2578.14 (2425.16, 2739.62) | -55.51 (-63.77, -47.26) |
| Turkmenistan | 986.43 (930.56, 1043.81) | 836.54 (790.72, 884.51) | -15.2 (-23.22, -7.17) |
| Tuvalu | 1.77 (1.68, 1.86) | 1.12 (1.06, 1.18) | -36.72 (-44.35, -29.09) |
| Uganda | 27176.45 (25602.97, 28852.07) | 28998.16 (27402.34, 30643.71) | 6.7 (-1.48, 14.89) |
| Ukraine | 3829.6 (3612.67, 4058.12) | 1492.27 (1410.42, 1580.89) | -61.03 (-69.19, -52.88) |
| United Arab Emirates | 190.14 (178.96, 201.15) | 235.54 (223.13, 248.16) | 23.88 (15.98, 31.77) |
| United Kingdom | 1885.9 (1871.67, 1899.12) | 1848.36 (1835.1, 1861.44) | -1.99 (-3.01, -0.97) |
| United Republic of Tanzania | 23306.34 (21963.48, 24676.54) | 26167.26 (24600.42, 27935.4) | 12.28 (3.64, 20.91) |
| United States Virgin Islands | 17.15 (16.31, 18.13) | 5.03 (4.77, 5.33) | -70.67 (-78.25, -63.09) |
| United States of America | 15133.5 (14313.31, 16011.04) | 11580.76 (10922.47, 12208.55) | -23.48 (-31.37, -15.58) |
| Uruguay | 323.54 (305.37, 342.57) | 188.57 (178.99, 199.99) | -41.72 (-49.72, -33.71) |
| Uzbekistan | 6024.32 (5703.18, 6360.2) | 6896.37 (6540.03, 7299.7) | 14.48 (6.72, 22.23) |
| Vanuatu | 40.35 (37.81, 42.77) | 56.28 (52.98, 59.62) | 39.48 (30.95, 48.01) |
| Venezuela (Bolivarian Republic of) | 3579.37 (3385.97, 3796.94) | 2556.09 (2406.56, 2708.49) | -28.59 (-36.82, -20.35) |
| Viet Nam | 23109.91 (21781.36, 24602.35) | 9612.93 (9089.45, 10146.16) | -58.4 (-66.62, -50.19) |
| Yemen | 4910.18 (4619.7, 5215.25) | 4790.79 (4502.04, 5057.75) | -2.43 (-10.82, 5.96) |
| Zambia | 6663.86 (6297.29, 7028.59) | 7175.64 (6765.17, 7602.75) | 7.68 (-0.33, 15.69) |
| Zimbabwe | 3891.32 (3634.77, 4144.38) | 4346.14 (4101.93, 4619.61) | 11.69 (2.84, 20.54) |
