# Supplementary material for: Analysis of global, regional, and national burdens of neonatal encephalopathy from 1990 to 2021: insights from the Global Burden of Disease Study 2021
Source: Front Public Health. 2025 Oct 8;13:1627448. doi: 10.3389/fpubh.2025.1627448 (PMC12540312; doi:10.3389/fpubh.2025.1627448)
Supplement: Supplementary file 4 [file Table_4.doc]

| Location | Incidence | | |
| --- | --- | --- | --- |
|  | Number (95% UI) | | Percentage  change  (95% UI) |
|  | 1990 | 2021 |  |
| Global | 1295891.1 (1276552.88, 1314238.82) | 1061448.15 (1047815.17, 1076766.11) | -18.09 (-20.08, -16.1) |
| Sex | | | |
| Female | 524054.79 (511319.84, 538318.18) | 423674.1 (414555.77, 433311.94) | -19.15 (-22.55, -15.76) |
| Male | 771836.32 (759824.14, 785417.65) | 637774.05 (629258.91, 647596.35) | -17.37 (-19.56, -15.17) |
| Low SDI | 359501.59 (353998.27, 365439.49) | 446383.14 (440080.38, 452996.39) | 24.17 (24.15, 24.19) |
| Low-middle SDI | 373971.25 (365035.12, 383072.25) | 282401.57 (276561.19, 288193.15) | -24.49 (-24.52, -24.45) |
| Middle SDI | 385710.35 (376861.6, 394205.18) | 230358.64 (225988.96, 234687.71) | -40.28 (-40.31, -40.25) |
| High-middle SDI | 125918.65 (121814.26, 129927.47) | 65901.22 (64248.82, 67714.72) | -47.66 (-47.71, -47.62) |
| High SDI | 49728.3 (48755.22, 50765.01) | 35552.71 (34750.29, 36257.78) | -28.51 (-28.54, -28.48) |
| Central Asia | 16732.97 (16319.36, 17128.51) | 17040.54 (16592.05, 17508.07) | 1.84 (1.8, 1.87) |
| Central Europe | 11520.8 (11321.87, 11729.76) | 5674.28 (5577.81, 5774.59) | -50.75 (-50.77, -50.72) |
| Eastern Europe | 16628.97 (16014.16, 17258.51) | 8903.54 (8543.71, 9288.43) | -46.46 (-46.51, -46.4) |
| High-income | 52146.49 (51228.54, 53163.51) | 36469.51 (35760.37, 37137.54) | -30.06 (-30.09, -30.04) |
| Australasia | 1153.13 (1105.87, 1201.74) | 905.4 (869.49, 942.65) | -21.48 (-21.54, -21.43) |
| High-income Asia Pacific | 9846.59 (9631.39, 10071.08) | 5319.44 (5243.68, 5397.56) | -45.98 (-46, -45.95) |
| High-income North America | 16421.23 (15583.49, 17318.14) | 12605.79 (11957.2, 13254.67) | -23.23 (-23.31, -23.16) |
| Southern Latin America | 8272.4 (7908.45, 8621.54) | 5316.42 (5115.54, 5537.61) | -35.73 (-35.79, -35.67) |
| Western Europe | 16453.13 (16204.56, 16711.47) | 12322.46 (12106.63, 12514.4) | -25.11 (-25.13, -25.08) |
| Andean Latin America | 12199.82 (11774.26, 12671.53) | 8893.49 (8568.76, 9233.6) | -27.1 (-27.15, -27.05) |
| Caribbean | 11251.67 (10938.45, 11590.63) | 8960.14 (8662.77, 9279.98) | -20.37 (-20.41, -20.32) |
| Central Latin America | 63585.9 (62862.27, 64391.19) | 33952.08 (33557.54, 34387.07) | -46.6 (-46.62, -46.59) |
| Tropical Latin America | 17373.82 (17065.85, 17673.75) | 13659.04 (13462.38, 13872.53) | -21.38 (-21.4, -21.36) |
| North Africa and Middle East | 61980.65 (60748, 63244.94) | 51158.96 (50111.71, 52229.04) | -17.46 (-17.49, -17.43) |
| East Asia | 203912.02 (192211.86, 215072.05) | 77307.32 (73099.67, 81366.54) | -62.09 (-62.17, -62.01) |
| Oceania | 1329.87 (1277.96, 1385.73) | 2221.46 (2119.81, 2332.09) | 67.04 (66.98, 67.11) |
| South Asia | 299012.38 (286263.03, 312398.83) | 227084.23 (217770.06, 237510.51) | -24.06 (-24.12, -23.99) |
| Southeast Asia | 146309.64 (141947.97, 150240.72) | 76278.96 (74288.03, 78391.07) | -47.86 (-47.9, -47.83) |
| Sub-Saharan Africa | 381906.11 (376290.96, 387984.31) | 493844.6 (486574.87, 502313.89) | 29.31 (29.29, 29.33) |
| Central Sub-Saharan Africa | 35404.09 (33901.87, 37012.07) | 45465.54 (43659.9, 47537.63) | 28.42 (28.36, 28.48) |
| Eastern Sub-Saharan Africa | 205640.37 (201048.29, 210675.03) | 226839.37 (222010.52, 231484.29) | 10.31 (10.28, 10.34) |
| Southern Sub-Saharan Africa | 17296.46 (16968.79, 17661.68) | 16788.72 (16429.07, 17167.1) | -2.94 (-2.97, -2.91) |
| Western Sub-Saharan Africa | 123565.18 (120189.9, 126780.35) | 204750.98 (198934.03, 210467.74) | 65.7 (65.66, 65.74) |
