# Supplementary material for: Analysis of global, regional, and national burdens of neonatal encephalopathy from 1990 to 2021: insights from the Global Burden of Disease Study 2021
Source: Front Public Health. 2025 Oct 8;13:1627448. doi: 10.3389/fpubh.2025.1627448 (PMC12540312; doi:10.3389/fpubh.2025.1627448)
Supplement: Supplementary file 5 [file Table_5.doc]

| Location | Mortality | | |
| --- | --- | --- | --- |
|  | ASIR (95% UI) | | EAPC  (95% CI) |
|  | 1990 | 2021 |  |
| Afghanistan | 29.12 (21.24,37.08) | 15.65 (11.66,20) | -1.74 (-1.82,-1.65) |
| Albania | 3.45 (2.49,4.54) | 1.64 (0.97,2.43) | -2.42 (-2.54,-2.3) |
| Algeria | 6.01 (3.89,9.21) | 1.76 (1.1,2.66) | -3.66 (-3.82,-3.5) |
| American Samoa | 4.17 (3.3,5.4) | 2.36 (1.6,3.23) | -1.84 (-2.08,-1.59) |
| Andorra | 1.1 (0.76,1.49) | 0.14 (0.07,0.21) | -4.87 (-5.33,-4.41) |
| Angola | 32.7 (23.98,42.3) | 17.35 (12.67,22.25) | -2.04 (-2.2,-1.89) |
| Antigua and Barbuda | 2.57 (2.05,3.12) | 1.07 (0.89,1.28) | -2.45 (-2.81,-2.09) |
| Argentina | 4.17 (3.67,4.66) | 0.95 (0.72,1.22) | -4.89 (-5.14,-4.65) |
| Armenia | 3.55 (2.93,4.39) | 1.13 (0.91,1.4) | -4.54 (-5.17,-3.92) |
| Australia | 1.2 (1.1,1.3) | 0.81 (0.66,0.99) | -0.62 (-1.34,0.1) |
| Austria | 1.33 (1.21,1.46) | 0.65 (0.55,0.77) | -1.28 (-1.94,-0.61) |
| Azerbaijan | 11.36 (8.79,14.62) | 8.48 (6.38,10.87) | -0.91 (-1.43,-0.38) |
| Bahamas | 6.07 (4.82,7.41) | 2.21 (1.63,2.84) | -2.94 (-3.31,-2.57) |
| Bahrain | 2.23 (1.57,2.9) | 0.46 (0.36,0.58) | -4.7 (-5.02,-4.38) |
| Bangladesh | 23.28 (16.33,29.94) | 12.76 (8.68,17.89) | -1.27 (-1.75,-0.79) |
| Barbados | 7.07 (5.77,8.44) | 3.02 (2.07,4.15) | -2.18 (-2.47,-1.88) |
| Belarus | 1.14 (0.87,1.44) | 0.24 (0.18,0.32) | -4.61 (-4.89,-4.33) |
| Belgium | 1.92 (1.74,2.12) | 1.06 (0.85,1.31) | -1.54 (-1.93,-1.15) |
| Belize | 7.39 (6.26,8.63) | 2.93 (2.33,3.64) | -2.82 (-3.09,-2.55) |
| Benin | 30.43 (23.14,40.07) | 22.67 (16.99,29.86) | -0.75 (-0.89,-0.61) |
| Bermuda | 2.99 (2.45,3.63) | 0.91 (0.59,1.29) | -3.03 (-3.45,-2.6) |
| Bhutan | 26.94 (18.99,35.75) | 11.69 (7.73,16.27) | -2.97 (-3.2,-2.74) |
| Bolivia (Plurinational State of) | 10.42 (7.71,13.47) | 4.06 (2.57,5.7) | -2.91 (-3.01,-2.8) |
| Bosnia and Herzegovina | 3.94 (2.34,5.75) | 1.08 (0.75,1.53) | -4.78 (-5.35,-4.2) |
| Botswana | 14.17 (9.62,21.19) | 10.42 (7.01,15.91) | -0.85 (-0.99,-0.72) |
| Brazil | 7.54 (6.74,8.31) | 2.7 (2.17,3.36) | -2.96 (-3.23,-2.69) |
| Brunei Darussalam | 1.48 (0.93,2.34) | 1.28 (0.89,1.84) | -0.07 (-0.27,0.13) |
| Bulgaria | 2.12 (1.89,2.4) | 0.82 (0.67,0.97) | -3.42 (-4.06,-2.77) |
| Burkina Faso | 20.25 (15.3,25.63) | 15.28 (11.03,19.94) | -0.59 (-0.7,-0.47) |
| Burundi | 20.16 (14.69,26.8) | 15.63 (11.11,22.75) | -0.68 (-0.96,-0.39) |
| Cabo Verde | 12.46 (9.47,15.57) | 5.48 (3.65,7.84) | -2.44 (-2.82,-2.07) |
| Cambodia | 12.03 (8.09,18.08) | 6.6 (4,9.77) | -2.15 (-2.34,-1.96) |
| Cameroon | 24.35 (18.37,30.71) | 15.84 (12.08,20.47) | -1.13 (-1.23,-1.03) |
| Canada | 1.34 (1.24,1.45) | 1.08 (0.89,1.3) | -0.63 (-0.83,-0.43) |
| Central African Republic | 33.43 (24.66,43.06) | 29.54 (21.69,38.8) | -0.19 (-0.31,-0.07) |
| Chad | 32.12 (24.94,39.03) | 27.45 (20.92,35.7) | -0.42 (-0.49,-0.36) |
| Chile | 3.16 (2.91,3.46) | 0.64 (0.53,0.77) | -3.63 (-4.18,-3.07) |
| China | 12.53 (10.3,14.92) | 1.75 (1.44,2.11) | -6.81 (-7.33,-6.28) |
| Colombia | 9.12 (7.86,10.35) | 1.71 (1.16,2.48) | -4.89 (-5.23,-4.55) |
| Comoros | 24.52 (17.61,31.76) | 16.09 (10.31,22.45) | -1.31 (-1.57,-1.04) |
| Congo | 19.02 (13.23,25.06) | 11.25 (6.75,15.81) | -1.62 (-1.94,-1.31) |
| Cook Islands | 3.81 (2.12,5.59) | 0.64 (0.32,1.12) | -7.62 (-8.31,-6.93) |
| Costa Rica | 2.81 (2.43,3.23) | 1.37 (1.08,1.7) | -2.33 (-2.61,-2.04) |
| Côte d'Ivoire | 26.65 (20.01,33.48) | 20.98(15.3,28.94) | -0.58(-0.77, -0.38) |
| Croatia | 3.01 (2.7,3.34) | 0.95 (0.73,1.22) | -2.67 (-3.29,-2.05) |
| Cuba | 2.92 (2.59,3.21) | 0.72 (0.61,0.84) | -4.36 (-4.69,-4.03) |
| Cyprus | 2.5 (1.66,3.37) | 0.36 (0.26,0.49) | -6.33 (-6.63,-6.04) |
| Czechia | 1.92 (1.71,2.11) | 0.43 (0.33,0.53) | -4.47 (-4.94,-3.99) |
| Democratic People's Republic of Korea | 7.23 (4.45,10.47) | 2.37 (1.2,3.71) | -3.28 (-3.69,-2.88) |
| Democratic Republic of the Congo | 22.75 (16.37,28.91) | 17.48 (12.59,24) | -0.15 (-0.48,0.17) |
| Denmark | 1.64 (1.43,1.87) | 0.69 (0.54,0.86) | -2.46 (-2.67,-2.26) |
| Djibouti | 14.33 (9.9,19.3) | 9.87 (6.76,13.39) | -1.15 (-1.45,-0.85) |
| Dominica | 7.2 (5.29,9.87) | 9.85 (6.27,14.19) | 1.67 (1.27,2.08) |
| Dominican Republic | 10.74 (8.16,13.79) | 4.44 (2.97,6.46) | -2.5 (-2.7,-2.3) |
| Ecuador | 5.64 (4.65,6.8) | 1.65 (1.28,2.12) | -4.15 (-4.73,-3.57) |
| Egypt | 1.31 (0.73,2.16) | 0.26 (0.18,0.36) | -4.86 (-5.34,-4.37) |
| El Salvador | 5.71 (4.57,7.29) | 1.21 (0.85,1.73) | -4.65 (-4.79,-4.52) |
| Equatorial Guinea | 25.48 (17.8,32.69) | 11.01 (4.83,16.84) | -2.91 (-3.13,-2.7) |
| Eritrea | 15.42 (10.78,21.94) | 12.35 (8.41,17.54) | -0.57 (-0.7,-0.44) |
| Estonia | 4.13 (3.75,4.58) | 0.18 (0.16,0.21) | -10.34 (-10.62,-10.05) |
| Eswatini | 12.97 (8.64,19.37) | 8.27 (5.54,12.37) | -1.07 (-1.23,-0.9) |
| Ethiopia | 35.49 (27.41,46.93) | 20.65 (16,26.6) | -1.7 (-1.89,-1.51) |
| Fiji | 6.43 (4.74,8.85) | 3.75 (2.23,5.46) | -1.88 (-2.06,-1.7) |
| Finland | 0.79 (0.69,0.9) | 0.35 (0.29,0.43) | -2.42 (-2.58,-2.26) |
| France | 1.31 (1.2,1.43) | 0.77 (0.61,0.95) | -1.61 (-1.94,-1.29) |
| Gabon | 18.65 (11.8,24.56) | 10.74 (6.19,15.46) | -1.24 (-1.54,-0.94) |
| Gambia | 31.19 (23.57,39.76) | 18.67 (12.92,26.45) | -1.58 (-1.73,-1.42) |
| Georgia | 21.3 (18.02,24.89) | 1.27 (0.99,1.63) | -10.35 (-11.67,-9) |
| Germany | 0.86 (0.77,0.96) | 0.69 (0.58,0.8) | -0.02 (-0.25,0.22) |
| Ghana | 33.81 (23.12,41.39) | 17.74 (11.17,25.94) | -1.7 (-1.87,-1.53) |
| Greece | 0.92 (0.82,1.01) | 0.45 (0.37,0.55) | -1.37 (-2.62,-0.1) |
| Greenland | 4.25 (3.17,5.46) | 1.16 (0.88,1.5) | -4.26 (-4.46,-4.07) |
| Grenada | 7.34 (5.94,9.09) | 3.78 (2.96,4.75) | -1.42 (-1.65,-1.19) |
| Guam | 2.95 (2.27,3.57) | 2.19 (1.53,2.99) | -0.46 (-0.69,-0.23) |
| Guatemala | 4.68 (3.89,5.64) | 2.86 (2.17,3.7) | -1.02 (-1.51,-0.53) |
| Guinea | 32.72 (25.97,41.2) | 19.23 (14.03,26.08) | -1.74 (-1.85,-1.63) |
| Guinea-Bissau | 33.67 (24.96,44.63) | 21.43 (15.45,30.61) | -1.38 (-1.64,-1.12) |
| Guyana | 13.17 (11.02,15.67) | 4.53 (3.21,6.29) | -3.13 (-3.51,-2.75) |
| Haiti | 15.72 (11.25,20.91) | 14.06 (10.2,19.31) | -0.21 (-0.3,-0.11) |
| Honduras | 7.7 (5.61,10.06) | 3.09 (1.99,4.46) | -2.9 (-3.21,-2.59) |
| Hungary | 2.01 (1.86,2.14) | 0.35 (0.27,0.44) | -5.36 (-5.87,-4.85) |
| Iceland | 1.04 (0.88,1.19) | 0.27 (0.22,0.33) | -3.93 (-4.18,-3.68) |
| India | 15.08 (11.79,21.71) | 7.4 (5.12,12.09) | -2.32 (-2.46,-2.19) |
| Indonesia | 11.87 (7.83,15.18) | 5.85 (3.78,7.81) | -2.36 (-2.52,-2.19) |
| Iran (Islamic Republic of) | 6.02 (4.65,8) | 0.47 (0.33,0.64) | -5.97 (-6.67,-5.26) |
| Iraq | 7.77 (5.54,10.92) | 2.5 (1.72,3.67) | -3.58 (-3.84,-3.33) |
| Ireland | 1.09 (0.97,1.21) | 0.63 (0.51,0.75) | -1.38 (-1.83,-0.93) |
| Israel | 0.91 (0.81,1.05) | 0.28 (0.23,0.34) | -2.69 (-3.1,-2.28) |
| Italy | 2.16 (2.07,2.24) | 0.47 (0.37,0.57) | -4.7 (-5.2,-4.2) |
| Jamaica | 6.12 (4.94,7.47) | 3.5 (2.49,4.81) | -1.37 (-1.63,-1.1) |
| Japan | 0.76 (0.72,0.8) | 0.23 (0.2,0.26) | -3.73 (-4.09,-3.36) |
| Jordan | 4.12 (2.9,5.79) | 1.14 (0.72,1.62) | -3.88 (-4.08,-3.69) |
| Kazakhstan | 3.24 (2.68,3.81) | 1.08 (0.89,1.34) | -3.24 (-4.1,-2.38) |
| Kenya | 13.17 (9.78,18.97) | 10.88 (8.25,13.84) | -0.38 (-0.52,-0.24) |
| Kiribati | 13.19 (9.04,17.94) | 7.11 (4.72,10.12) | -1.98 (-2.02,-1.95) |
| Kuwait | 3.21 (2.74,3.81) | 0.49 (0.39,0.62) | -4.97 (-5.74,-4.19) |
| Kyrgyzstan | 10.84 (8.42,13.04) | 3.21 (2.69,3.76) | -3.8 (-4.6,-3) |
| Lao People's Democratic Republic | 20.23 (13.13,30) | 10.14 (5.65,15.83) | -2.42 (-2.62,-2.22) |
| Latvia | 5.42 (4.87,5.98) | 1.27 (1.04,1.52) | -5.15 (-5.7,-4.59) |
| Lebanon | 2.62 (1.73,3.83) | 0.48 (0.32,0.71) | -5.46 (-5.71,-5.21) |
| Lesotho | 20.56 (14.85,28.61) | 15.74 (11.16,21.62) | -0.86 (-0.95,-0.77) |
| Liberia | 38.02 (29.54,47.58) | 18.6 (12.39,27.65) | -2.18 (-2.35,-2.02) |
| Libya | 3.55 (2.19,5.73) | 1.28 (0.68,2.05) | -2.95 (-3.48,-2.42) |
| Lithuania | 3.59 (3.23,3.99) | 0.4 (0.34,0.47) | -7.66 (-8.05,-7.28) |
| Luxembourg | 2.15 (1.9,2.42) | 0.58 (0.48,0.71) | -3.83 (-4.43,-3.22) |
| Madagascar | 7.91 (6.02,10.13) | 6.78 (4.21,9.67) | -0.21 (-0.43,0) |
| Malawi | 24.6 (18.26,31.22) | 13.71 (10.01,17.91) | -1.75 (-1.98,-1.52) |
| Malaysia | 2.77 (2.06,3.67) | 0.99 (0.68,1.34) | -2.59 (-3.14,-2.03) |
| Maldives | 11.78 (8.38,17.16) | 2.96 (2.14,4.1) | -4.28 (-4.43,-4.13) |
| Mali | 42.91 (32.3,54.31) | 24.44 (18.56,32.13) | -1.79 (-1.95,-1.63) |
| Malta | 1.29 (1.11,1.48) | 0.66 (0.51,0.85) | -1.5 (-1.85,-1.15) |
| Marshall Islands | 6.28 (3.35,9.44) | 3.66 (2.06,5.82) | -1.79 (-2.03,-1.55) |
| Mauritania | 23.26 (16.61,30) | 12.61 (9.09,17.53) | -1.79 (-2.03,-1.55) |
| Mauritius | 6.05 (5.37,6.77) | 1.87 (1.49,2.21) | -3.24 (-3.74,-2.73) |
| Mexico | 8.5 (7.76,9.3) | 2.34 (1.88,2.84) | -3.97 (-4.15,-3.79) |
| Micronesia (Federated States of) | 7.62 (3.71,11.66) | 3.1 (1.45,5.16) | -3.05 (-3.18,-2.93) |
| Monaco | 1.83 (1.27,2.54) | 0.67 (0.54,0.82) | -3.56 (-3.74,-3.38) |
| Mongolia | 18.35 (14.23,23.34) | 5.6 (4.15,7.45) | -4.19 (-4.59,-3.79) |
| Montenegro | 7.73 (6.33,9.48) | 1.25 (0.92,1.71) | -6.18 (-6.77,-5.58) |
| Morocco | 6.08 (3.72,9.22) | 1.65 (0.99,2.62) | -4.08 (-4.37,-3.79) |
| Mozambique | 30.79 (18.42,40.19) | 17.85 (10.53,25.51) | -1.48 (-1.61,-1.35) |
| Myanmar | 11.98 (7.98,18.11) | 7 (4.12,10.98) | -1.76 (-2,-1.52) |
| Namibia | 13.93 (9.49,20.24) | 7.92 (5.43,11.22) | -1.38 (-1.57,-1.19) |
| Nauru | 5.36 (2.73,8.34) | 3.71 (2.09,6.08) | -1.24 (-1.7,-0.77) |
| Nepal | 35.9 (27.05,44.37) | 16.01 (11.5,21.69) | -2.53 (-2.73,-2.33) |
| Netherlands | 1.83 (1.67,2) | 0.84 (0.71,0.98) | -2.4 (-2.61,-2.19) |
| New Zealand | 1.57 (1.42,1.72) | 0.47 (0.4,0.54) | -3.57 (-3.98,-3.16) |
| Nicaragua | 8.85 (6.9,10.95) | 2.82 (2.02,3.92) | -3.69 (-3.78,-3.61) |
| Niger | 21.33 (15.69,27.13) | 15.2 (11.32,20.01) | -1.55 (-1.75,-1.35) |
| Nigeria | 33.55 (28.22,43.61) | 25.4 (19.95,31.38) | -0.73 (-0.84,-0.61) |
| Niue | 4.23 (2.37,6.14) | 6.26 (4.09,9.6) | -0.6 (-1.25,0.05) |
| North Macedonia | 4.23 (3.22,5.2) | 0.63 (0.47,0.79) | -5.13 (-5.54,-4.72) |
| Northern Mariana Islands | 1.72 (1.19,2.32) | 1.12 (0.78,1.57) | -0.53 (-0.89,-0.17) |
| Norway | 3.18 (2.93,3.42) | 0.91 (0.79,1.05) | -3.66 (-4.05,-3.28) |
| Oman | 1.47 (0.85,2.65) | 0.27 (0.17,0.41) | -4.53 (-5.21,-3.85) |
| Pakistan | 34.52 (27.78,42.87) | 32.21 (24.64,40.19) | -0.44 (-0.61,-0.27) |
| Palau | 6.92 (5.02,9.47) | 3.96 (2.95,5.13) | -1.52 (-1.72,-1.31) |
| Palestine | 5.27 (4.02,6.94) | 1.44 (1,1.92) | -3.76 (-4.02,-3.49) |
| Panama | 3.9 (3.28,4.63) | 1.64 (1.24,2.11) | -2.22 (-2.43,-2.02) |
| Papua New Guinea | 5.74 (3.96,7.84) | 5.14 (3.51,7.27) | -0.3 (-0.43,-0.17) |
| Paraguay | 5.77 (4.13,7.8) | 2.7 (1.76,3.79) | -2.72 (-2.96,-2.47) |
| Peru | 11.28 (8.58,14.44) | 3.6 (2.33,5.07) | -2.86 (-3.12,-2.61) |
| Philippines | 6.98 (5.42,8.75) | 4.01 (3.06,5.18) | -1.32 (-1.49,-1.15) |
| Poland | 2.84 (2.6,3.14) | 0.25 (0.2,0.32) | -8.34 (-8.73,-7.95) |
| Portugal | 2.62 (2.44,2.8) | 0.68 (0.57,0.79) | -3.07 (-3.95,-2.19) |
| Puerto Rico | 1.39 (1.22,1.59) | 0.45 (0.36,0.55) | -3.03 (-3.55,-2.51) |
| Qatar | 0.91 (0.62,1.25) | 0.18 (0.12,0.26) | -4.77 (-4.89,-4.66) |
| Republic of Korea | 1.3 (1.01,1.66) | 0.25 (0.18,0.33) | -4.4 (-4.69,-4.11) |
| Republic of Moldova | 10.76 (9.24,12.41) | 1.62 (1.2,2.17) | -6.51 (-6.89,-6.13) |
| Romania | 3.36 (2.92,3.83) | 0.44 (0.38,0.5) | -7.28 (-7.69,-6.87) |
| Russian Federation | 7.44 (7.13,7.77) | 0.77 (0.68,0.85) | -7.54 (-7.92,-7.16) |
| Rwanda | 22.2 (17.19,27.83) | 11.75 (8.38,16.21) | -2.06 (-2.28,-1.85) |
| Saint Kitts and Nevis | 16.06 (14.12,18.39) | 6.16 (4.77,7.83) | -2.67 (-2.91,-2.43) |
| Saint Lucia | 6.22 (5.04,7.45) | 4.64 (3.3,6.35) | -0.41 (-0.69,-0.12) |
| Saint Vincent and the Grenadines | 6.34 (5.05,7.86) | 2.1 (1.57,2.81) | -3.34 (-3.68,-3.01) |
| Samoa | 6.4 (2.74,10.74) | 2.8 (1.23,4.83) | -2.58 (-2.67,-2.5) |
| San Marino | 3.16 (2.33,4.11) | 0.49 (0.3,0.77) | -5.11 (-5.36,-4.85) |
| Sao Tome and Principe | 12.75 (9.09,16.88) | 5.13 (3.45,7.5) | -2.72 (-3.21,-2.23) |
| Saudi Arabia | 5.91 (4.22,7.98) | 0.41 (0.27,0.57) | -8.63 (-8.88,-8.37) |
| Senegal | 23.94 (16.94,33.37) | 14.53 (10.32,20.68) | -1.49 (-1.72,-1.25) |
| Serbia | 7.82 (5.82,9.56) | 1.1 (0.86,1.35) | -6.88 (-7.47,-6.28) |
| Seychelles | 3.66 (2.57,5.09) | 2.23 (1.31,3.69) | -1.05 (-1.29,-0.81) |
| Sierra Leone | 32.74 (24.54,41.82) | 23.63 (17,31.4) | -0.82 (-1.01,-0.63) |
| Singapore | 0.93 (0.83,1.05) | 0.22 (0.17,0.27) | -4.03 (-4.58,-3.48) |
| Slovakia | 1.5 (1.16,1.84) | 0.46 (0.33,0.61) | -4 (-4.32,-3.67) |
| Slovenia | 1.16 (1.03,1.3) | 0.13 (0.11,0.15) | -6.86 (-7.12,-6.6) |
| Solomon Islands | 8.25 (5.72,10.93) | 4.63 (3.12,6.46) | -1.83 (-1.98,-1.68) |
| Somalia | 19.73 (12.99,31.65) | 19.99 (13.59,31.97) | 0.24 (0.07,0.41) |
| South Africa | 12.89 (10.64,15.7) | 7.88 (6.05,10.13) | -1.86 (-2.06,-1.67) |
| South Sudan | 21.88 (14.58,29.56) | 28.85 (19.18,40.47) | 1.01 (0.93,1.09) |
| Spain | 1.52 (1.4,1.65) | 0.63 (0.52,0.74) | -2.12 (-2.61,-1.63) |
| Sri Lanka | 4.95 (3.77,6.44) | 1.11 (0.78,1.46) | -5.13 (-5.49,-4.78) |
| Sudan | 10.09 (7,15.23) | 3.29 (1.95,4.97) | -3.42 (-3.62,-3.21) |
| Suriname | 11.94 (8.98,15.58) | 6.2 (4.31,9.04) | -2.23 (-2.37,-2.1) |
| Sweden | 1.34 (1.23,1.46) | 0.57 (0.5,0.65) | -1.93 (-2.29,-1.56) |
| Switzerland | 1.05 (0.93,1.2) | 1.03 (0.83,1.23) | 0.3 (-0.05,0.65) |
| Syrian Arab Republic | 7.08 (4.17,10.79) | 1.43 (0.81,2.37) | -4.12 (-4.8,-3.43) |
| Taiwan (Province of China) | 0.22 (0.2,0.25) | 0.37 (0.3,0.43) | 3.31 (2.41,4.23) |
| Tajikistan | 8.98 (6.23,11.58) | 8.04 (5.66,10.95) | -0.34 (-0.5,-0.19) |
| Thailand | 4.01 (2.78,5.38) | 0.86 (0.65,1.08) | -4.86 (-5.11,-4.61) |
| Timor-Leste | 13.89 (9,21.46) | 7.48 (4.42,11.28) | -2.14 (-2.2,-2.07) |
| Togo | 27.84 (21.48,34.79) | 17.72 (12.51,24.33) | -1.25 (-1.37,-1.14) |
| Tokelau | 4.56 (2.48,6.94) | 8.95 (5.27,13.15) | -2.1 (-3.65,-0.52) |
| Tonga | 1.39 (0.77,2.26) | 0.58 (0.31,0.93) | -2.67 (-2.78,-2.56) |
| Trinidad and Tobago | 7.14 (5.71,8.69) | 2.43 (1.76,3.26) | -3.38 (-3.6,-3.16) |
| Tunisia | 5.46 (3.63,7.95) | 1.06 (0.7,1.6) | -5.02 (-5.11,-4.92) |
| Turkey | 5.19 (3.45,7.21) | 1 (0.71,1.37) | -4.8 (-5.11,-4.49) |
| Turkmenistan | 11.52 (9.49,13.38) | 8.28 (5.96,10.61) | -0.62 (-0.95,-0.28) |
| Tuvalu | 12.86 (6.87,18.72) | 3.62 (1.89,5.63) | -3.94 (-4.1,-3.78) |
| Uganda | 25.19 (19.67,30.71) | 15.18 (10.9,19.86) | -1.51 (-1.73,-1.28) |
| Ukraine | 2.88 (2.31,3.53) | 1.22 (0.96,1.5) | -2.03 (-2.45,-1.61) |
| United Arab Emirates | 0.99 (0.61,1.61) | 0.18 (0.12,0.25) | -4.52 (-4.85,-4.19) |
| United Kingdom | 1.62 (1.55,1.69) | 0.57 (0.48,0.66) | -2.91 (-3.18,-2.64) |
| United Republic of Tanzania | 13.18 (8.21,20.36) | 8.44 (4.1,14.48) | -1.2 (-1.66,-0.73) |
| United States of America | 1.19 (1.15,1.23) | 0.79 (0.7,0.88) | -0.97 (-1.11,-0.83) |
| United States Virgin Islands | 3.6 (2.55,4.79) | 0.92 (0.61,1.31) | -4.01 (-4.22,-3.79) |
| Uruguay | 3.98 (3.57,4.44) | 0.67 (0.5,0.85) | -5.61 (-5.95,-5.26) |
| Uzbekistan | 17.29 (14.91,20.03) | 4.65 (3.79,5.64) | -3.91 (-4.88,-2.92) |
| Vanuatu | 7.25 (3.07,12.94) | 4.48 (1.87,8.12) | -1.68 (-1.86,-1.49) |
| Venezuela (Bolivarian Republic of) | 5.14 (4.56,5.74) | 3.66 (2.65,4.93) | -0.73 (-1.22,-0.24) |
| Viet Nam | 5.35 (3.62,7.3) | 1.6 (1.02,2.37) | -3.85 (-3.94,-3.77) |
| Yemen | 9.02 (5.83,15.78) | 3.84 (2.35,6.2) | -2.79 (-2.9,-2.68) |
| Zambia | 19.57 (15.69,24.23) | 12.86 (8.86,18.15) | -0.99 (-1.37,-0.62) |
| Zimbabwe | 7.49 (5.04,10.48) | 8.26 (4.98,12.42) | 0.92 (0.65,1.18) |
