# Supplementary material for: Analysis of global, regional, and national burdens of neonatal encephalopathy from 1990 to 2021: insights from the Global Burden of Disease Study 2021
Source: Front Public Health. 2025 Oct 8;13:1627448. doi: 10.3389/fpubh.2025.1627448 (PMC12540312; doi:10.3389/fpubh.2025.1627448)
Supplement: Supplementary file 6 [file Table_6.doc]

| Location | Mortality | | |
| --- | --- | --- | --- |
|  | ASMR (95% UI) | | EAPC  (95% CI) |
|  | 1990 | 2021 |  |
| Global | 13.81 (12.65, 15.71) | 9.75  (8.26,11.71) | -1.23(-1.36, -1.11) |
| Sex | | | |
| Female | 11.86 (10.52,13.7) | 8.34  (6.95,9.94,) | -1.28(-1.41, -1.15) |
| Male | 15.63 (13.97,17.96) | 11.07  (9.15,13.21) | -1.20(-1.33, -1.07) |
| Low SDI | 23.94 (21.02,28.73) | 17.77 (14.77,21.58) | -0.79 (-0.91,-0.68) |
| Low-middle SDI | 17.86 (15.66,21.5) | 12.07 (9.9,14.49) | -1.28 (-1.42,-1.14) |
| Middle SDI | 11.5 (10.14,12.89) | 4.06 (3.42,4.86) | -3.48 (-3.66,-3.29) |
| High-middle SDI | 6.79 (6.1,7.66) | 1.09 (0.93,1.27) | -6.17 (-6.46,-5.87) |
| High SDI | 1.6 (1.51,1.71) | 0.66 (0.58,0.72) | -2.58 (-2.68,-2.48) |
| Central Asia | 12.09 (10.92,13.32) | 4.64 (3.91,5.51) | -3.06 (-3.69,-2.42) |
| Central Europe | 3.22 (2.93,3.47) | 0.5 (0.43,0.59) | -6.43 (-6.67,-6.19) |
| Eastern Europe | 6.08 (5.81,6.33) | 0.82 (0.73,0.91) | -6.52 (-6.87,-6.17) |
| High-income | 1.49 (1.45,1.53) | 0.69 (0.61,0.77) | -2.11 (-2.25,-1.96) |
| Australasia | 1.27 (1.18,1.37) | 0.75 (0.62,0.91) | -1.01 (-1.61,-0.41) |
| High-income Asia Pacific | 0.95 (0.85,1.08) | 0.24 (0.21,0.26) | -4.08 (-4.37,-3.79) |
| High-income North America | 1.2 (1.17,1.24) | 0.82 (0.73,0.91) | -0.92 (-1.05,-0.8) |
| Southern Latin America | 3.87 (3.52,4.22) | 0.85 (0.67,1.07) | -4.66 (-4.89,-4.42) |
| Western Europe | 1.47 (1.43,1.52) | 0.65 (0.56,0.74) | -2.19 (-2.4,-1.98) |
| Andean Latin America | 9.67 (7.97,11.57) | 3.18 (2.32,4.11) | -3.1 (-3.29,-2.9) |
| Caribbean | 9.38 (7.93,11.13) | 8.03 (6.11,10.63) | -0.32 (-0.44,-0.19) |
| Central Latin America | 7.72 (7.2,8.34) | 2.42 (1.92,3.07) | -3.45 (-3.64,-3.25) |
| Tropical Latin America | 7.47 (6.69,8.22) | 2.7 (2.18,3.34) | -2.95 (-3.22,-2.69) |
| North Africa and Middle East | 6.51 (5.52,8.1) | 2.9 (2.28,3.56) | -2.8 (-2.93,-2.67) |
| East Asia | 12.24 (10.06,14.55) | 1.75 (1.44,2.11) | -6.71 (-7.22,-6.19) |
| Oceania | 6.01 (4.61,7.8) | 4.94 (3.49,6.75) | -0.62 (-0.74,-0.49) |
| South Asia | 18.95 (15.74,25.06) | 12.94 (10.23,16.84) | -1.25 (-1.36,-1.14) |
| Southeast Asia | 8.94 (6.66,10.94) | 4.52 (3.21,5.63) | -2.2 (-2.34,-2.06) |
| Sub-Saharan Africa | 25.83 (23.3,29.59) | 18.53 (15.23,22.32) | -0.91 (-1.05,-0.77) |
| Central Sub-Saharan Africa | 25.04 (19.08,31.43) | 17.66 (13.95,22.43) | -0.67 (-0.92,-0.42) |
| Eastern Sub-Saharan Africa | 23.37 (19.98,28.27) | 15.39 (12.33,19.21) | -1.27 (-1.45,-1.09) |
| Southern Sub-Saharan Africa | 11.92 (10.07,14.2) | 8.28 (6.39,10.82) | -1.15 (-1.29,-1.02) |
| Western Sub-Saharan Africa | 31.06 (27.02,37.69) | 22.11 (17.98,26.42) | -0.95 (-1.06,-0.84) |
