# Supplementary material for: Analysis of global, regional, and national burdens of neonatal encephalopathy from 1990 to 2021: insights from the Global Burden of Disease Study 2021
Source: Front Public Health. 2025 Oct 8;13:1627448. doi: 10.3389/fpubh.2025.1627448 (PMC12540312; doi:10.3389/fpubh.2025.1627448)
Supplement: Supplementary file 7 [file Table_7.doc]

| Location | Mortality | | |
| --- | --- | --- | --- |
|  | Number (95% UI) | | Percentage  change  (95% UI) |
|  | 1990 | 2021 |  |
| Afghanistan | 6086.73 (4435.19, 7751.59) | 32.49 (23.88, 42.63) | -99.47 (-139.14, -59.8) |
| Albania | 134.25 (97.04, 176.78) | 3.76 (2.67, 5.14) | -97.2 (-141.47, -52.92) |
| Algeria | 2218.64 (1436.4, 3401.34) | 770.45 (483.71, 1164.45) | -65.27 (-127.83, -2.72) |
| American Samoa | 3.51 (2.78, 4.55) | 1093.19 (770.17, 1489.09) | 31045.01 (31003.63, 31086.4) |
| Andorra | 0.28 (0.19, 0.38) | 1815.54 (1077.6, 2739.97) | 648307.14 (648249.52, 648364.77) |
| Angola | 7780.57 (5728.06, 10057.99) | 4.25 (3.14, 5.47) | -99.95 (-138.86, -61.03) |
| Antigua and Barbuda | 1.51 (1.2, 1.83) | 36 (28.45, 44.64) | 2284.11 (2253.5, 2314.72) |
| Argentina | 1390.58 (1225.58, 1556.09) | 4375.21 (3336.73, 5654.43) | 214.63 (185.6, 243.66) |
| Armenia | 128.84 (106.59, 159.49) | 408.7 (310.49, 529.24) | 217.22 (183.49, 250.94) |
| Australia | 148.29 (136.37, 160.87) | 9.99 (6.64, 14.24) | -93.26 (-132.21, -54.32) |
| Austria | 59.33 (53.96, 65.45) | 2.46 (1.92, 3.08) | -95.85 (-121.64, -70.07) |
| Azerbaijan | 1009.79 (780.78, 1299.24) | 1164 (754.43, 1642.23) | 15.27 (-30.7, 61.24) |
| Bahamas | 16.11 (12.77, 19.65) | 41.37 (32.43, 52.12) | 156.8 (124.79, 188.8) |
| Bahrain | 14.21 (10, 18.53) | 213.61 (175.25, 264.03) | 1403.24 (1366.68, 1439.79) |
| Bangladesh | 47181.46 (33069.33, 60716.61) | 4577.03 (3340.83, 6208.24) | -90.3 (-133.19, -47.41) |
| Barbados | 14.16 (11.56, 16.9) | 455.33 (304.66, 661.88) | 3115.61 (3072.1, 3159.12) |
| Belarus | 78.31 (59.35, 98.46) | 1130.59 (684.95, 1672.81) | 1343.74 (1293.41, 1394.06) |
| Belgium | 115.66 (104.94, 127.99) | 23884.7 (17208.71, 32797.16) | 20550.79 (20516.67, 20584.91) |
| Belize | 22.35 (18.94, 26.1) | 840.31 (573.14, 1189.15) | 3659.78 (3619.76, 3699.79) |
| Benin | 3451.29 (2619.48, 4546.55) | 85.83 (56.82, 128.77) | -97.51 (-147.87, -47.16) |
| Bermuda | 1.28 (1.05, 1.55) | 687.47 (412.17, 965.71) | 53608.59 (53563.68, 53653.51) |
| Bhutan | 286.5 (201.81, 380.18) | 0.05 (0.03, 0.09) | -99.98 (-184.34, -15.63) |
| Bolivia (Plurinational State of) | 1137.1 (840.41, 1470.71) | 3.06 (1.95, 4.41) | -99.73 (-148.68, -50.78) |
| Bosnia and Herzegovina | 128.17 (76.21, 186.82) | 12.32 (8.78, 16.42) | -90.39 (-143.54, -37.24) |
| Botswana | 315 (213.81, 471.27) | 22.36 (14.89, 31.96) | -92.9 (-148.8, -37.01) |
| Brazil | 11672.87 (10435.95, 12878.25) | 10.3 (8.41, 12.35) | -99.91 (-121.79, -78.03) |
| Brunei Darussalam | 5.02 (3.16, 7.91) | 17.4 (12.61, 23.29) | 246.61 (190.3, 302.92) |
| Bulgaria | 102.03 (90.74, 115.16) | 1.9 (1.55, 2.3) | -98.14 (-121.1, -75.17) |
| Burkina Faso | 4477.44 (3376.09, 5669.05) | 8594.18 (6402.12, 11312.71) | 91.94 (53.58, 130.31) |
| Burundi | 2505.56 (1824.79, 3331.07) | 3349.06 (2379.38, 4768.74) | 33.67 (-12.98, 80.31) |
| Cabo Verde | 75.09 (57.05, 93.8) | 21.94 (12.97, 32.46) | -70.78 (-121.48, -20.08) |
| Cambodia | 2460.81 (1651.92, 3705.44) | 4931.37 (3542.41, 6681.18) | 100.4 (47.92, 152.87) |
| Cameroon | 5553.95 (4185.65, 7009.8) | 12439.13 (9440.26, 16359.45) | 123.97 (86.29, 161.65) |
| Canada | 261.57 (241.81, 283.89) | 0.52 (0.29, 0.85) | -99.8 (-153.18, -46.42) |
| Central African Republic | 2013.65 (1486.1, 2582.12) | 1.72 (1.33, 2.19) | -99.91 (-136.92, -62.91) |
| Chad | 4865.94 (3784.79, 5898.84) | 9509.32 (6937.42, 13118.07) | 95.43 (56.34, 134.52) |
| Chile | 463.91 (426.74, 506.79) | 26.85 (22.38, 31.31) | -94.21 (-112.96, -75.47) |
| China | 138507.07 (113865.19, 164943.52) | 11469.71 (8232.14, 15009.46) | -91.72 (-126.55, -56.89) |
| Colombia | 4032.25 (3472.62, 4575.75) | 0.08 (0.05, 0.11) | -100 (-150.87, -49.12) |
| Comoros | 231.2 (165.88, 299.56) | 26.49 (18.39, 38.59) | -88.54 (-136.37, -40.71) |
| Congo | 830.02 (577.71, 1091.66) | 335.39 (169.67, 525.23) | -59.59 (-120.98, 1.79) |
| Cook Islands | 0.81 (0.45, 1.19) | 501.11 (444.04, 555.89) | 61765.43 (61718.12, 61812.74) |
| Costa Rica | 109.91 (95.36, 126.67) | 71.97 (61.13, 83.56) | -34.52 (-55.63, -13.41) |
| Croatia | 80.43 (72.23, 89.37) | 10.32 (6.45, 15.68) | -87.17 (-133.05, -41.28) |
| Cuba | 251.15 (222.84, 276.07) | 254.44 (198.28, 327.37) | 1.31 (-26.18, 28.8) |
| Cyprus | 16.54 (10.96, 22.3) | 18.08 (14.63, 21.93) | 9.31 (-30.43, 49.05) |
| Czechia | 119.4 (106.3, 130.74) | 5260.7 (3499.16, 7377.57) | 4305.95 (4267.69, 4344.2) |
| Côte d'Ivoire | 7046.22 (5282.54, 8858.28) | 135.68 (76.61, 225.34) | -98.07 (-158.47, -37.68) |
| Democratic People's Republic of Korea | 1863.07 (1145.9, 2698.28) | 17136.33 (11653.23, 24022.68) | 819.79 (764.67, 874.91) |
| Democratic Republic of the Congo | 19510.26 (14050.98, 24739.85) | 556.27 (376.21, 805.01) | -97.15 (-144.43, -49.86) |
| Denmark | 50.24 (43.86, 57.3) | 2122.85 (1706.99, 2576.46) | 4125.42 (4100.96, 4149.88) |
| Djibouti | 106.8 (73.85, 143.71) | 817.96 (589.6, 1137.46) | 665.88 (619.07, 712.69) |
| Dominica | 6.45 (4.74, 8.84) | 1.14 (0.95, 1.35) | -82.33 (-118.57, -46.08) |
| Dominican Republic | 1153.71 (876.08, 1483.04) | 4.22 (3.13, 5.8) | -99.63 (-140.74, -58.53) |
| Ecuador | 814.92 (671.29, 983.08) | 11.45 (9.1, 13.53) | -98.59 (-125.8, -71.39) |
| Egypt | 1175.46 (656.01, 1939.25) | 143 (97.98, 194) | -87.83 (-151.91, -23.75) |
| El Salvador | 476.11 (381.28, 608.09) | 167.36 (109.27, 234.97) | -64.85 (-109.32, -20.37) |
| Equatorial Guinea | 249.86 (174.46, 320.22) | 32.49 (23.03, 45.17) | -87 (-131.86, -42.14) |
| Eritrea | 1091.05 (762.69, 1552.59) | 62.74 (51.9, 74.87) | -94.25 (-134.82, -53.68) |
| Estonia | 42.89 (38.93, 47.54) | 999.87 (689.02, 1466.51) | 2231.24 (2191.08, 2271.4) |
| Eswatini | 200.74 (133.67, 299.84) | 35.63 (27.97, 43.83) | -82.25 (-129.26, -35.25) |
| Ethiopia | 40782.38 (31529.45, 53651.27) | 1190.32 (763.83, 1766.62) | -97.08 (-147.18, -46.98) |
| Fiji | 58.27 (42.97, 80.13) | 173.71 (124.21, 241.42) | 198.11 (151.69, 244.53) |
| Finland | 24.89 (21.81, 28.32) | 18.45 (12.28, 27.09) | -25.87 (-68.1, 16.36) |
| France | 485.17 (444.32, 528.64) | 1401.53 (1240.58, 1561.28) | 188.87 (174.51, 203.24) |
| Gabon | 317.94 (201.26, 418.76) | 6941.23 (5007.25, 9061.13) | 2083.19 (2038.22, 2128.16) |
| Gambia | 669.07 (504.23, 855.4) | 27.36 (21.36, 35.2) | -95.91 (-132.35, -59.47) |
| Georgia | 885.15 (748.85, 1034.43) | 3.39 (2.28, 4.79) | -99.62 (-139.98, -59.25) |
| Germany | 367.58 (326.46, 408.79) | 9360.84 (7720.97, 11293.55) | 2446.61 (2424.49, 2468.74) |
| Ghana | 9757.14 (6673.64, 11948.1) | 3726.11 (2860.99, 4788.49) | -61.81 (-99.22, -24.4) |
| Greece | 46.31 (41.35, 51.11) | 15.15 (11.76, 18.71) | -67.29 (-92.49, -42.08) |
| Greenland | 2.46 (1.83, 3.16) | 1780.75 (1451.72, 2162.22) | 72288.21 (72254.57, 72321.85) |
| Grenada | 8.51 (6.9, 10.55) | 43.66 (35.16, 51.99) | 413.04 (384.24, 441.85) |
| Guam | 5.36 (4.13, 6.47) | 736.42 (530.82, 1051.8) | 13639.18 (13597.56, 13680.8) |
| Guatemala | 774.56 (644.01, 932.69) | 21.99 (16.34, 29.54) | -97.16 (-132.51, -61.81) |
| Guinea | 4540.96 (3599.78, 5712.94) | 0.46 (0.24, 0.72) | -99.99 (-156.17, -43.81) |
| Guinea-Bissau | 736.88 (546.32, 977.87) | 18.82 (15.09, 23.26) | -97.45 (-133.87, -61.02) |
| Guyana | 164.63 (137.81, 195.85) | 1449.88 (965.86, 2155.38) | 780.69 (736.04, 825.34) |
| Haiti | 1890.1 (1348.18, 2521.55) | 2.62 (1.87, 3.6) | -99.86 (-145.1, -54.62) |
| Honduras | 661.01 (481.3, 863.32) | 7870.02 (6003.85, 10171.1) | 1090.61 (1051.41, 1129.8) |
| Hungary | 121.4 (112.76, 129.51) | 3602.58 (2120.91, 5647.41) | 2867.53 (2818.1, 2916.96) |
| Iceland | 2.32 (1.97, 2.65) | 161.21 (114.1, 212.4) | 6848.71 (6815, 6882.41) |
| India | 181139.43 (141657.57, 260769.87) | 14.48 (9.99, 20.5) | -99.99 (-148.95, -51.03) |
| Indonesia | 26702.07 (17598.57, 34141.06) | 102.22 (84.92, 119.1) | -99.62 (-134.82, -64.42) |
| Iran (Islamic Republic of) | 4500.01 (3473.88, 5973.86) | 11.46 (8.57, 14.63) | -99.75 (-138.14, -61.35) |
| Iraq | 2567 (1829.97, 3613.01) | 55.84 (42.14, 71.57) | -97.82 (-141.43, -54.22) |
| Ireland | 28.65 (25.46, 31.85) | 0.76 (0.52, 1.05) | -97.35 (-135.14, -59.56) |
| Israel | 46.31 (40.94, 52.94) | 2079.4 (1482.88, 2869.9) | 4390.17 (4354.4, 4425.95) |
| Italy | 582.76 (557.35, 605.45) | 438.41 (315.76, 561.96) | -24.77 (-53.15, 3.61) |
| Jamaica | 169.43 (136.68, 206.8) | 91.27 (61.05, 127.32) | -46.13 (-87.93, -4.33) |
| Japan | 458.14 (435.92, 478.61) | 546.45 (411, 700.72) | 19.28 (-7.64, 46.19) |
| Jordan | 262.18 (184.44, 368.84) | 3.96 (3.19, 4.9) | -98.49 (-139.85, -57.13) |
| Kazakhstan | 576.43 (476.81, 677.75) | 8.32 (3.65, 14.37) | -98.56 (-165.19, -31.92) |
| Kenya | 6201.95 (4604.56, 8929.18) | 34.84 (29.65, 40.92) | -99.44 (-137.88, -60.99) |
| Kiribati | 16.97 (11.64, 23.09) | 10041.82 (7333.66, 12877.47) | 59073.95 (59030.37, 59117.54) |
| Kuwait | 52.51 (44.8, 62.33) | 115.47 (94.9, 140.62) | 119.9 (94.01, 145.79) |
| Kyrgyzstan | 688.62 (534.89, 828.04) | 7624.66 (3697.19, 13074.29) | 1007.24 (942.17, 1072.31) |
| Lao People's Democratic Republic | 1667.28 (1080.56, 2481.23) | 8.2 (6.68, 9.96) | -99.51 (-146.06, -52.95) |
| Latvia | 96.23 (86.52, 106.09) | 241.37 (202.26, 282.45) | 150.83 (131.35, 170.3) |
| Lebanon | 107.06 (70.75, 156.36) | 1.14 (0.98, 1.32) | -98.94 (-141.81, -56.06) |
| Lesotho | 527.18 (380.64, 733.46) | 3724.04 (2567.52, 5257.95) | 606.41 (557.17, 655.65) |
| Liberia | 2035.87 (1579.02, 2553.98) | 4311.63 (3466.87, 5354.6) | 111.78 (79.34, 144.23) |
| Libya | 222.1 (136.91, 358.62) | 161.14 (127.18, 198.32) | -27.45 (-82.02, 27.13) |
| Lithuania | 97.38 (87.68, 108.51) | 3408.29 (2453.46, 4528.92) | 3399.99 (3367.72, 3432.26) |
| Luxembourg | 5.21 (4.61, 5.85) | 28.02 (22.91, 32.82) | 437.81 (416.41, 459.22) |
| Madagascar | 1971.47 (1497.13, 2524.83) | 6.56 (4.44, 9.21) | -99.67 (-144.48, -54.86) |
| Malawi | 5867.98 (4353.13, 7424.06) | 5.73 (4.3, 7.22) | -99.9 (-136.31, -63.5) |
| Malaysia | 661.05 (491.68, 876.84) | 1.32 (0.98, 1.76) | -99.8 (-141.4, -58.2) |
| Maldives | 50.12 (35.66, 73.01) | 2676.67 (1964.92, 3515) | 5240.52 (5193.33, 5287.72) |
| Mali | 9110.2 (6861.91, 11537.09) | 489.81 (346.33, 669.33) | -94.62 (-136.4, -52.84) |
| Malta | 3.45 (2.99, 3.97) | 2.01 (1.13, 3.19) | -41.74 (-95.34, 11.86) |
| Marshall Islands | 4.52 (2.41, 6.78) | 320.87 (220.86, 444.01) | 6998.89 (6939.51, 7058.27) |
| Mauritania | 963.11 (687.27, 1242.86) | 9080.45 (6763.77, 11602.76) | 842.83 (803.56, 882.09) |
| Mauritius | 67.17 (59.66, 75.17) | 9440.28 (5575.47, 13492.6) | 13954.31 (13910.81, 13997.81) |
| Mexico | 10347.73 (9450.03, 11320.43) | 18.84 (7.88, 34.15) | -99.82 (-170.1, -29.53) |
| Micronesia (Federated States of) | 11.54 (5.62, 17.66) | 3740.25 (2728.64, 4884.73) | 32311.18 (32251.6, 32370.76) |
| Monaco | 0.24 (0.16, 0.33) | 0.44 (0.33, 0.56) | 83.33 (41.13, 125.54) |
| Mongolia | 634.95 (492.43, 807) | 4.58 (3.9, 5.34) | -99.28 (-128.68, -69.88) |
| Montenegro | 37.05 (30.35, 45.46) | 237.03 (167.25, 320.26) | 539.76 (501.59, 577.92) |
| Morocco | 2295.62 (1402.19, 3482.34) | 49.56 (26.48, 79.45) | -97.84 (-167.89, -27.79) |
| Mozambique | 8892.45 (5338.6, 11604.25) | 9179.22 (6239.4, 14660.74) | 3.22 (-54.61, 61.06) |
| Myanmar | 6404.4 (4265.8, 9685.77) | 12.15 (8.18, 17.78) | -99.81 (-157.71, -41.91) |
| Namibia | 342.22 (233.13, 497.42) | 257.69 (204.09, 317.43) | -24.7 (-69.14, 19.74) |
| Nauru | 0.91 (0.46, 1.41) | 69.96 (46.25, 97.42) | 7587.91 (7524.6, 7651.23) |
| Nepal | 13483.73 (10159.87, 16667.5) | 0.83 (0.44, 1.33) | -99.99 (-159.43, -40.56) |
| Netherlands | 174.36 (159.35, 190.27) | 205.85 (152.62, 273.82) | 18.06 (-12.69, 48.81) |
| New Zealand | 45.49 (41.15, 50.01) | 0.32 (0.23, 0.45) | -99.3 (-137.31, -61.28) |
| Nicaragua | 603.42 (470.25, 746.99) | 11280.35 (8616, 14673.32) | 1769.4 (1734.09, 1804.71) |
| Niger | 4505.97 (3334.41, 5734.51) | 2.78 (1.3, 4.63) | -99.94 (-165.52, -34.36) |
| Nigeria | 65281.63 (54928.59, 84950.67) | 2301.68 (1669.04, 3160.26) | -96.47 (-136.2, -56.75) |
| Niue | 0.09 (0.05, 0.14) | 31.56 (27.34, 36.04) | 34966.67 (34920.98, 35012.35) |
| North Macedonia | 69 (52.54, 84.9) | 58.36 (46.8, 72.32) | -15.42 (-47.49, 16.65) |
| Northern Mariana Islands | 1.01 (0.7, 1.36) | 1789.85 (1092.4, 2887.38) | 177112.87 (177052.85, 177172.89) |
| Norway | 91.07 (84.01, 98.03) | 83.35 (58.36, 111.61) | -8.48 (-41.33, 24.37) |
| Oman | 50.77 (29.43, 91.32) | 6164.88 (4674.1, 7841.34) | 12042.76 (11976.61, 12108.91) |
| Pakistan | 69350.97 (55804.89, 86109.31) | 92.07 (72.7, 111.66) | -99.87 (-130.28, -69.45) |
| Palau | 1.04 (0.75, 1.42) | 242.5 (163.29, 370.38) | 23217.31 (23163.92, 23270.69) |
| Palestine | 221.4 (168.47, 292.37) | 0.07 (0.05, 0.11) | -99.97 (-162.57, -37.37) |
| Panama | 110.54 (93.07, 131.3) | 0.54 (0.45, 0.65) | -99.51 (-124.58, -74.45) |
| Papua New Guinea | 404.26 (278.72, 552.77) | 22.86 (18.69, 27.08) | -94.35 (-132.89, -55.8) |
| Paraguay | 357.95 (256.14, 484.59) | 100304.59 (78778.05, 123896.27) | 27921.96 (27882.91, 27961) |
| Peru | 3477.72 (2643.63, 4451.89) | 0.61 (0.49, 0.74) | -99.98 (-132.35, -67.62) |
| Philippines | 6760.55 (5249.53, 8472.34) | 192.7 (159.49, 220.21) | -97.15 (-125.72, -68.58) |
| Poland | 748.5 (684.6, 825.84) | 21.15 (16.72, 26.4) | -97.17 (-121.93, -72.42) |
| Portugal | 145.12 (135.46, 155.32) | 507.58 (304.65, 803.84) | 249.77 (200.12, 299.41) |
| Puerto Rico | 43.8 (38.32, 50.21) | 853.98 (475.96, 1333.17) | 1849.73 (1797.74, 1901.72) |
| Qatar | 4.96 (3.39, 6.86) | 475.04 (301.64, 667.78) | 9477.42 (9425.25, 9529.59) |
| Republic of Korea | 420.96 (325.07, 536.98) | 795.57 (575.18, 1071.86) | 88.99 (48.89, 129.09) |
| Republic of Moldova | 410.78 (352.46, 473.74) | 146.31 (86.42, 220.63) | -64.38 (-112.57, -16.2) |
| Romania | 489.98 (426.03, 558.73) | 17 (13.74, 20.32) | -96.53 (-120.16, -72.9) |
| Russian Federation | 7102.52 (6809.29, 7412.17) | 0.34 (0.26, 0.44) | -100 (-129.13, -70.86) |
| Rwanda | 3363.4 (2603.71, 4213.15) | 3.82 (2.62, 5.24) | -99.89 (-141.77, -58) |
| Saint Kitts and Nevis | 7.18 (6.31, 8.22) | 34390.98 (26637.99, 44305.53) | 478883.01 (478854.05, 478911.97) |
| Saint Lucia | 10.66 (8.63, 12.77) | 218.07 (149.41, 308.94) | 1945.68 (1904.24, 1987.13) |
| Saint Vincent and the Grenadines | 7.84 (6.24, 9.72) | 2061.82 (1455.22, 2831.41) | 26198.72 (26158.61, 26238.83) |
| Samoa | 16.59 (7.1, 27.85) | 131.11 (83.98, 182.98) | 690.3 (617.28, 763.32) |
| San Marino | 0.37 (0.27, 0.48) | 191.91 (157.88, 230.33) | 51767.57 (51735.04, 51800.09) |
| Sao Tome and Principe | 28.18 (20.05, 37.31) | 3499.69 (2487.75, 5094.08) | 12319.06 (12270.86, 12367.25) |
| Saudi Arabia | 1447.85 (1033.73, 1955.29) | 8239.15 (5187.6, 12047.68) | 469.06 (416.66, 521.46) |
| Senegal | 3949.38 (2789.06, 5509.07) | 2.79 (1.95, 3.81) | -99.93 (-147.64, -52.22) |
| Serbia | 517.39 (385.39, 632.53) | 0.03 (0.02, 0.05) | -99.99 (-169.56, -30.43) |
| Seychelles | 2.9 (2.03, 4.03) | 224.25 (169.83, 280.84) | 7632.76 (7590.32, 7675.19) |
| Sierra Leone | 3157.37 (2367.84, 4043.53) | 38.02 (33.03, 42.89) | -98.8 (-128.34, -69.25) |
| Singapore | 22.5 (20.03, 25.28) | 1155.77 (786.68, 1641.24) | 5036.76 (4997.99, 5075.52) |
| Slovakia | 57.32 (44.5, 70.37) | 0.32 (0.21, 0.46) | -99.44 (-142.57, -56.32) |
| Slovenia | 12.42 (11.08, 13.91) | 22.08 (17.14, 27.32) | 77.78 (52.05, 103.5) |
| Solomon Islands | 53.92 (37.42, 71.45) | 8.59 (6.19, 11.88) | -84.07 (-129.79, -38.35) |
| Somalia | 3637.21 (2396.55, 5823.51) | 0.07 (0.03, 0.12) | -100 (-173.18, -26.82) |
| South Africa | 6475.61 (5345.22, 7887.57) | 13.87 (11.75, 16.1) | -99.79 (-124.91, -74.66) |
| South Sudan | 2667.59 (1776.76, 3605.45) | 9.78 (7.28, 12.83) | -99.63 (-144.18, -55.08) |
| Spain | 292.18 (269.76, 317.21) | 5.76 (4.51, 7.11) | -98.03 (-121.91, -74.15) |
| Sri Lanka | 855.17 (651.7, 1111.8) | 95.65 (82.08, 110.46) | -88.82 (-119.54, -58.09) |
| Sudan | 4164.86 (2873.63, 6316.99) | 1.39 (1.07, 1.78) | -99.97 (-148.48, -51.46) |
| Suriname | 52.46 (39.43, 68.46) | 318.89 (226.09, 437.79) | 507.87 (464.65, 551.1) |
| Sweden | 79.91 (73.14, 87) | 25.17 (20.16, 30.51) | -68.5 (-90.82, -46.19) |
| Switzerland | 42.62 (37.6, 48.64) | 96081.87 (73472.28, 119862.65) | 225338.46 (225311.05, 225365.86) |
| Syrian Arab Republic | 1556.99 (918.37, 2376.57) | 690.57 (477.89, 978.86) | -55.65 (-114.88, 3.59) |
| Taiwan (Province of China) | 34.85 (31.25, 38.66) | 26.67 (22.47, 31.54) | -23.47 (-43.5, -3.44) |
| Tajikistan | 896.84 (623.03, 1157.05) | 77652.04 (53706.24, 126852.31) | 8558.41 (8502.69, 8614.12) |
| Thailand | 1998.98 (1388.87, 2683.7) | 221.86 (127.8, 319.44) | -88.9 (-142.89, -34.92) |
| Timor-Leste | 230.29 (148.97, 357.16) | 244.86 (185.35, 316.14) | 6.33 (-46.17, 58.83) |
| Togo | 2135.19 (1646.22, 2669.05) | 226.8 (157.19, 307.14) | -89.38 (-130.2, -48.56) |
| Tokelau | 0.08 (0.04, 0.12) | 45.26 (30.54, 63.14) | 56475 (56414.18, 56535.82) |
| Tonga | 2.17 (1.2, 3.54) | 2745.88 (1703.6, 3915.08) | 126438.25 (126370.73, 126505.77) |
| Trinidad and Tobago | 82 (65.69, 99.84) | 118.13 (74.34, 167.85) | 44.06 (-0.65, 88.78) |
| Tunisia | 574.96 (382.31, 836.59) | 0.1 (0.08, 0.13) | -99.98 (-144.08, -55.88) |
| Turkey | 3684.52 (2448.66, 5121.57) | 0.21 (0.14, 0.31) | -99.99 (-152.05, -47.94) |
| Turkmenistan | 693.2 (571.17, 805.16) | 32.64 (19.45, 47.55) | -95.29 (-141.54, -49.05) |
| Tuvalu | 2.11 (1.13, 3.08) | 12404.72 (8017.28, 16547.27) | 587801.42 (587743.64, 587859.21) |
| Uganda | 11032.4 (8600.28, 13476.65) | 198 (86.74, 302.7) | -98.21 (-157.05, -39.36) |
| Ukraine | 929.01 (747.03, 1140.2) | 67.78 (47.82, 96.9) | -92.7 (-134.64, -50.77) |
| United Arab Emirates | 23.06 (14.07, 37.47) | 55.61 (39.55, 76.29) | 141.15 (80.61, 201.7) |
| United Kingdom | 620.03 (593.15, 646.26) | 10.78 (8.59, 13.41) | -98.26 (-121.03, -75.49) |
| United Republic of Tanzania | 7570.68 (4707.13, 11685.94) | 11.83 (9.4, 14.9) | -99.84 (-151.44, -48.25) |
| United States Virgin Islands | 4.02 (2.84, 5.34) | 24.52 (21.18, 28.12) | 509.95 (475.69, 544.21) |
| United States of America | 2384.89 (2311.5, 2456.86) | 16.1 (12.41, 20.62) | -99.32 (-124.95, -73.7) |
| Uruguay | 107.05 (96.09, 119.24) | 261.74 (220.93, 304.69) | 144.5 (125.18, 163.82) |
| Uzbekistan | 5894.35 (5084.49, 6829.54) | 1848.85 (1114.22, 2780.71) | -68.63 (-116.07, -21.2) |
| Vanuatu | 21.12 (8.94, 37.7) | 3.78 (2.63, 5.46) | -82.1 (-159.78, -4.42) |
| Venezuela (Bolivarian Republic of) | 1357.07 (1203.29, 1514.78) | 5677.7 (4254.84, 7476.43) | 318.38 (287.78, 348.98) |
| Viet Nam | 4965.47 (3360.39, 6782.42) | 3.91 (3.04, 4.95) | -99.92 (-142.24, -57.6) |
| Yemen | 2805.29 (1797.62, 4926.64) | 1.69 (1, 2.81) | -99.94 (-177.12, -22.76) |
| Zambia | 3542.93 (2838.34, 4381.61) | 114.79 (76.85, 171.58) | -96.76 (-143.42, -50.1) |
| Zimbabwe | 1390.49 (935.12, 1946.89) | 329.55 (212.59, 476.26) | -76.3 (-130.37, -22.23) |
