# Supplementary material for: Analysis of global, regional, and national burdens of neonatal encephalopathy from 1990 to 2021: insights from the Global Burden of Disease Study 2021
Source: Front Public Health. 2025 Oct 8;13:1627448. doi: 10.3389/fpubh.2025.1627448 (PMC12540312; doi:10.3389/fpubh.2025.1627448)
Supplement: Supplementary file 8 [file Table_8.doc]

| Location | Mortality | | |
| --- | --- | --- | --- |
|  | Number (95% UI) | | Percentage  change  (95% UI) |
|  | 1990 | 2021 |  |
| Global | 883082.06 (809080.14, 1004097.14) | 603605.62 (511190.63, 725270.82) | -31.65 (-52.54, -10.76) |
| Sex | | | |
| Female | 365501.85 (324486.08, 422333.27) | 249299.44 (207717.77, 297288.92) | -31.79 (-54.2, -9.39) |
| Male | 517580.21 (462704.86, 594591.69) | 354306.18 (293053.95, 422742.43) | -31.55 (-53.85, -9.25) |
| Low SDI | 252433.87 (221890.83, 302642.24) | 6141.82 (5240.13, 7168.78) | -97.57 (-119.98, -75.15) |
| Low-middle SDI | 330761.6 (290033.13, 398249.79) | 306445.26 (254816.55, 372292.37) | -7.35 (-32.55, 17.85) |
| Middle SDI | 230185.33 (203006.57, 257985.36) | 62255.79 (52405.36, 74587.1) | -72.95 (-94.4, -51.51) |
| High-middle SDI | 59663.86 (53627.64, 67336.53) | 225205.61 (184776.5, 270362.88) | 277.46 (255.25, 299.66) |
| High SDI | 9643.22 (9090.06, 10301.5) | 3263.65 (2895.99, 3592.88) | -66.16 (-78.54, -53.77) |
| Central Asia | 11408.18 (10302.43, 12572.09) | 195871.57 (154828.91, 254921.15) | 1616.94 (1589.52, 1644.36) |
| Central Europe | 2659.86 (2424.62, 2867.55) | 100837.36 (80791.86, 125859.4) | 3691.08 (3667.23, 3714.93) |
| Eastern Europe | 8757.13 (8372.19, 9115.09) | 3063.74 (2331.64, 4052.83) | -65.01 (-93.42, -36.61) |
| High-income | 8994.33 (8746.03, 9238.64) | 3467.43 (3059.07, 3865.21) | -61.45 (-73.39, -49.51) |
| Australasia | 193.79 (180.61, 208.7) | 6469.2 (4997.51, 8456.44) | 3238.25 (3210.55, 3265.95) |
| High-income Asia Pacific | 906.62 (808.71, 1026.88) | 319.07 (249.72, 399.48) | -64.81 (-91.18, -38.44) |
| High-income North America | 2648.97 (2576.28, 2725.79) | 1593.9 (1418.59, 1772.34) | -39.83 (-51.28, -28.38) |
| Southern Latin America | 1961.63 (1785.08, 2140.91) | 37710.51 (29788.07, 47900.43) | 1822.41 (1796.74, 1848.08) |
| Western Europe | 3283.31 (3189.78, 3387.52) | 137.67 (122.74, 152.48) | -95.81 (-107.02, -84.59) |
| Andean Latin America | 5429.74 (4477.47, 6495.63) | 187983.41 (152955.27, 224574.3) | 3362.11 (3335.49, 3388.72) |
| Caribbean | 4044.3 (3420.78, 4802.24) | 710.05 (631.97, 791.72) | -82.44 (-102.89, -61.99) |
| Central Latin America | 18472.6 (17246.1, 19960.63) | 24371.69 (17335.39, 30366.05) | 31.93 (4.21, 59.66) |
| Tropical Latin America | 12030.82 (10782.02, 13234.95) | 4546.27 (3596.77, 5762.84) | -62.21 (-88.12, -36.3) |
| North Africa and Middle East | 34054.81 (28916.41, 42412.36) | 1287.45 (1116.04, 1454.62) | -96.22 (-120, -72.44) |
| East Asia | 140404.99 (115423.27, 166876.31) | 254.76 (215.12, 297.98) | -99.82 (-124.32, -75.32) |
| Oceania | 645.47 (495.17, 837.66) | 16645.26 (13107.24, 20435.51) | 2478.78 (2444.31, 2513.26) |
| South Asia | 311442.09 (258810.61, 411799.77) | 4478.99 (3613.78, 5534.65) | -98.56 (-131.17, -65.96) |
| Southeast Asia | 52902.8 (39416.4, 64777.76) | 1893.48 (1380.31, 2443.7) | -96.42 (-133.34, -59.5) |
| Sub-Saharan Africa | 271834.96 (245273.71, 311079.4) | 333000.49 (273827.54, 400981.21) | 22.5 (-0.1, 45.11) |
| Central Sub-Saharan Africa | 30702.3 (23438.12, 38413.81) | 4565.81 (3841.95, 5421.36) | -85.13 (-115.03, -55.23) |
| Eastern Sub-Saharan Africa | 99536.16 (85145.98, 120061.31) | 9724.25 (8006.35, 11756.9) | -90.23 (-116.3, -64.16) |
| Southern Sub-Saharan Africa | 9251.24 (7811.05, 11025.68) | 129.34 (107.33, 156.56) | -98.6 (-124.37, -72.83) |
| Western Sub-Saharan Africa | 132345.26 (115019.65, 160700.94) | 1011.83 (714.36, 1384.31) | -99.24 (-136.57, -61.9) |
